# TrmB Family Transcription Factor as a Thiol-Based Regulator of Oxidative Stress Response

**DOI:** 10.1128/mbio.00633-22

**Published:** 2022-07-20

**Authors:** Paula Mondragon, Sungmin Hwang, Lakshmi Kasirajan, Rebecca Oyetoro, Angelina Nasthas, Emily Winters, Ricardo L. Couto-Rodriguez, Amy Schmid, Julie A. Maupin-Furlow

**Affiliations:** a Department of Microbiology and Cell Science, Institute of Food and Agricultural Sciences, University of Florida, Gainesville, Florida, USA; b Department of Biology, Duke University, Durham, North Carolina, USA; c Center for Genomics and Computational Biology, Duke University, Durham, North Carolina, USA; d Genetics Institute, University of Florida, Gainesville, Florida, USA; e ICAR-Sugarcane Breeding Institute, Coimbatore, India; Pennsylvania State University

**Keywords:** archaea, DNA binding, TrmB family, oxidative stress, redox switch, systems biology, thiol chemistry, transcription factors

## Abstract

Oxidative stress causes cellular damage, including DNA mutations, protein dysfunction, and loss of membrane integrity. Here, we discovered that a TrmB (transcription regulator of *mal* operon) family protein (Pfam PF01978) composed of a single winged-helix DNA binding domain (InterPro IPR002831) can function as thiol-based transcriptional regulator of oxidative stress response. Using the archaeon Haloferax volcanii as a model system, we demonstrate that the TrmB-like OxsR is important for recovery of cells from hypochlorite stress. OxsR is shown to bind specific regions of genomic DNA, particularly during hypochlorite stress. OxsR-bound intergenic regions were found proximal to oxidative stress operons, including genes associated with thiol relay and low molecular weight thiol biosynthesis. Further analysis of a subset of these sites revealed OxsR to function during hypochlorite stress as a transcriptional activator and repressor. OxsR was shown to require a conserved cysteine (C24) for function and to use a CG-rich motif upstream of conserved BRE/TATA box promoter elements for transcriptional activation. Protein modeling suggested the C24 is located at a homodimer interface formed by antiparallel α helices, and that oxidation of this cysteine would result in the formation of an intersubunit disulfide bond. This covalent linkage may promote stabilization of an OxsR homodimer with the enhanced DNA binding properties observed in the presence of hypochlorite stress. The phylogenetic distribution TrmB family proteins, like OxsR, that have a single winged-helix DNA binding domain and conserved cysteine residue suggests this type of redox signaling mechanism is widespread in Archaea.

## INTRODUCTION

Oxidative stress can be exceedingly damaging to cells. Once the levels of reactive species overwhelm the antioxidant capacity of cells, lipid peroxidation, protein denaturation, DNA hydroxylation, and other damaging effects occur that impair cellular viability (1). To survive these challenges, cells must sense and respond to oxidant challenge, shifts in redox balance, and the damage encountered due to oxidative stress. Transcription factors (TFs) that sense and respond to reactive species are found to mediate global changes in gene expression to remedy the damage (2, 3). These TFs often have metalloclusters, cofactors, or residues (cysteine, histidine, or methionine) that are sensitive to oxidant and serve as “redox switches” (4, 5). These switches can turn TF function on or off in coordinating gene coexpression networks associated with electron flow, antioxidant systems, and other pathways (6–8).

Archaea have an array of metabolic strategies and physiological adaptations that enable them to sense and respond to extreme environments. TFs are central to sensing and responding to environmental cues and in relaying this signal to coordinate gene-expression networks. Thus, TFs are anticipated to be important in facilitating the ability of archaea to respond and thrive in extreme environments including exposure to reactive species (9, 10). TFs with redox switches are diverse and widespread in bacteria and likely to function in archaea to sense oxidant rich conditions. Archaea, while distinct from bacteria in their use a eukaryotic-like basal transcription machinery, have TFs that are often related to bacteria (9, 10). This similarity in TFs is due to their evolution which includes shared ancestral proteins and interdomain horizontal gene transfer events between archaea and bacteria. Thus, it is surprising that the bacterial TFs, such as OhrR (11–13), SarA/MgrA (14), PerR (15), HypR (16), YodB (17), QsrR (18), MosR (19), SarZ (20), OxyR (21–23), SoxR (24, 25), and FNR (26), that use redox switches to mediate global alterations of transcriptional networks, are not readily identified in archaea.

The two TFs that have been identified in archaea to use redox switches are from the ArsR family, MsvR (MTH_1349) and SurR (PF0095). ArsR proteins usually control metal transporters and respond to metal ions (9). However, the archaeal MsvR and SurR can sense redox state through the oxidation of key cysteine residues which results in reduced DNA binding activity (9). SurR responds to elemental sulfur (S^0^), an electron acceptor in the *Thermococcales*, leading to the inactivation of gene expression associated with H_2_ production and the derepression of genes needed for S^0^ metabolism (27–30). MsvR responds to oxidation resulting in the derepression of the transcription of itself and an adjacent operon implicated in the oxidative stress response (31–33). MsvR appears exclusive to methanogens, while SurR clusters to the Archaeal Clusters of Orthologous Gene (arCOG) group arCOG01684 (34), suggesting its general function is more widespread.

TrmB (transcription regulator of *mal* operon) family TFs, though not yet correlated with redox stress, are widespread in archaea and found in bacteria (9, 35). TrmB family proteins appear to have undergone an evolutionary expansion in archaea after divergence from bacteria, with homologs accounting for 12% of the total number of TFs in archaea compared with 0.5% in bacteria (9). TrmB family TFs have an N-terminal DNA binding domain that is sometimes fused to a C-terminal ligand sensing domain. The ligands that bind to the C-terminal domain can be sugars or other molecular factors. TrmB family TFs with these two domains (the N-terminal DNA binding domain and C-terminal ligand sensing domain) typically function as global transcriptional activators and/or repressors of sugar transport and metabolism including glycolysis, gluconeogenesis, the TCA cycle, amino acid metabolism, methanogenesis, and autotrophic pathways (36–46). TrmB homologs with the single DNA binding domain, while less characterized, appear to function differently than their two-domain counterparts. This variation is exemplified by TrmBL2 (TrmB-like protein 2), an abundant DNA binding protein of the *Thermococcales* that clusters to arCOG02037. TrmBL2 functions as a global transcriptional repressor and a chromatin binding protein that can rearrange genomic DNA from a conventional histone-bound “beads-on-a-string” architecture to a thick fibrous structure (34). Compared with other TrmB family proteins, TrmBL2 has an expanded function as it can bind single-stranded and double-stranded DNA (47).

Identification of TFs and other global regulatory systems used by archaea to sense and respond to reactive species is imperative to provide new insights into hypertolerance mechanisms. Haloarchaea provide a useful resource for this discovery as they inhabit some of the saltiest places on Earth, including hypersaline lakes, marine salterns, and brine inclusions, which are high in reactive species (48, 49). Compared with most organisms, haloarchaea display an order of magnitude higher tolerance to oxidant rich conditions, which is likely associated with their adaptation to these hypersaline ecosystems (50, 51). Haloarchaea employ an unusual “high-salt-in” strategy in which molar concentrations of potassium and chloride are accumulated intracellularly to maintain osmoprotection and cellular homeostasis (52–55). These high concentrations of chloride promote the generation of reactive chloride species (RCS), reactive oxygen species (ROS), and other stressful agents (56–58). High levels of reactive species are also generated in hypersaline habitats through common cycles of desiccation-rehydration and intense UV radiation (56, 57, 59). Surprisingly, haloarchaea thrive under these conditions and are often the last remaining communities when the salt concentrations reach saturation (60).

Regulators of oxidative stress responses are identified in haloarchaea; however, the mechanisms of how these regulators sense oxidant remains to be determined. Included among these factors are RosR (VNG0258H) and SHOxi. RosR, a PadR-type TF with a winged helix-turn-helix (wHTH) domain, is required for gene expression dynamics during extreme oxidative stress in *Halobacterium* sp. NRC-1 (61–63). RosR homologs cluster to the arCOG00006 group and are found widespread among haloarchaea suggesting a common mechanism, yet the residues that may directly sense oxidant are not readily identifiable. SHOxi is a small noncoding RNA in Haloferax volcanii that impacts redox balance by destabilizing malic enzyme mRNA and, thus, decreasing the ratio of NAD^+^/NADH (64). SHOxi is upregulated during oxidative stress, suggesting other factors serve upstream of this response to sense the redox status of cells.

Here, we discover that TrmB family proteins can function as TFs that sense redox status and facilitate the recovery of cells from hypochlorite stress. Using Haloferax volcanii as a model system, we targeted the TrmB family protein HVO_2970 of arCOG02242 for study, as it undergoes a several-fold increase in protein abundance after exposure of cells to hypochlorite stress as determined by stable isotope labeling of amino acids in cell culture (SILAC)-based proteomic analysis (65). Here, we reveal HVO_2970 is a thiol-based TF required for recovery of cells from hypochlorite stress and, thus, it is named OxsR, for oxidative stress responsive regulator. By coupling chromatin immunoprecipitation sequencing (ChIP-seq) and quantitative real-time PCR (qRT-PCR) analyses, we demonstrate that OxsR regulates the expression of genes associated with thiol relay, low molecular weight thiol synthesis, and other related functions during hypochlorite stress. OxsR functions as a transcriptional activator and repressor, with these distinctions found to correlate with the presence and positioning of a CG-rich motif relative to the BRE/TATA box promoter consensus sequence. By site-directed mutagenesis, a cysteine residue (C24) oriented at a predicted homodimer interface was demonstrated to be important for OxsR function. This cysteine residue was conserved among TrmB-like single DNA binding domain proteins from diverse archaeal phyla suggesting this type of redox signaling mechanism is widespread and represents a new type of thiol-based TF.

## RESULTS

### OxsR phylogenetic distribution.

**FIG 1:**
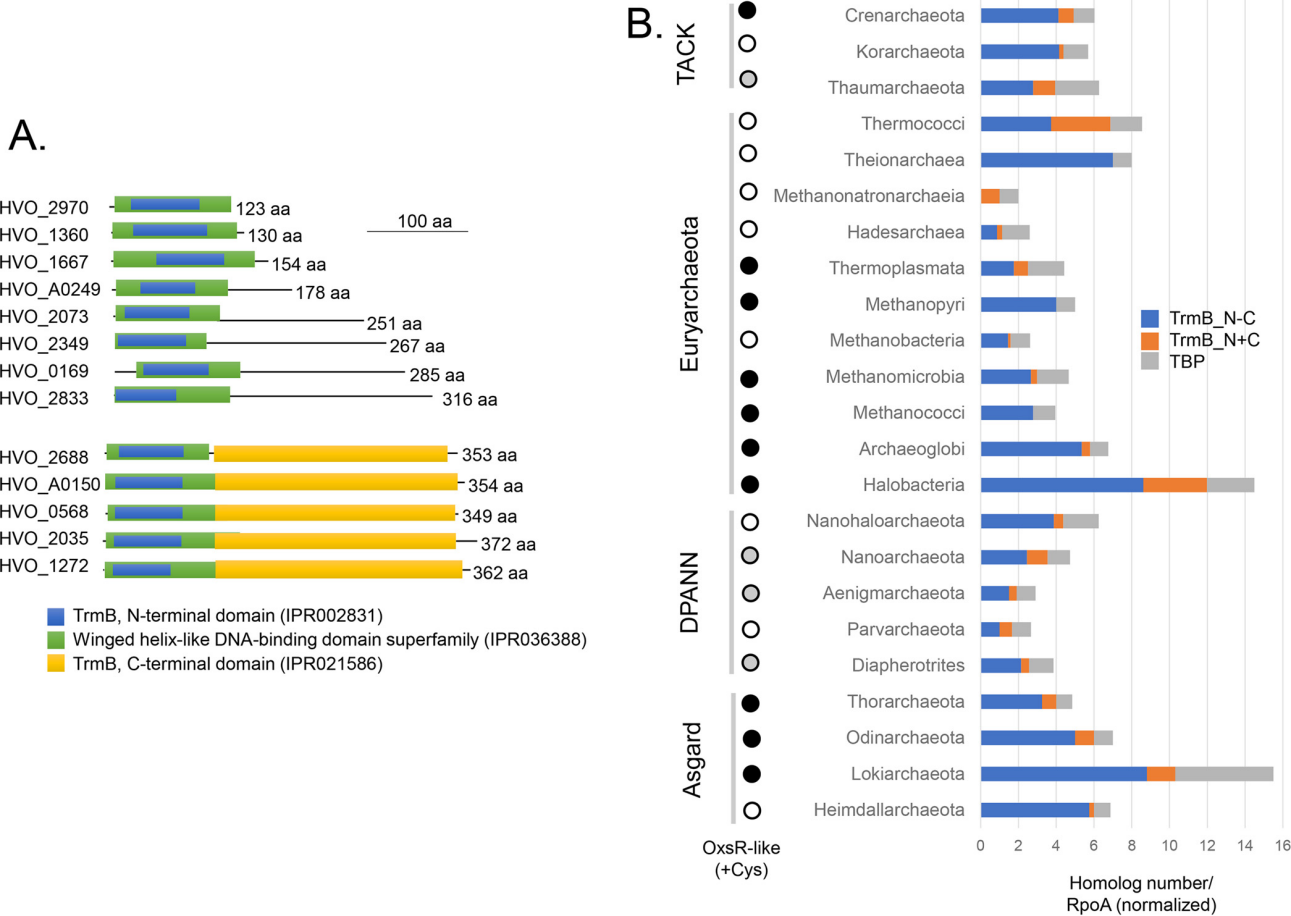
Haloferax volcanii OxsR (HVO_2970) is a member of the TrmB family and is conserved across multiple phyla of *Archaea*. (A) Two major types of TrmB family members observed. H. volcanii TrmB family proteins with: (i) a winged helix-turn-helix (wHTH) DNA binding domain (green and blue; OxsR is of this type); and (ii) an N-terminal wHTH DNA binding domain and C-terminal ligand (sugar) binding domain (yellow). (B) Distribution of OxsR and TrmB family homologs among archaea. TrmB family proteins with only an N-terminal DNA binding domain (IPR002831) (blue bar), TrmB proteins with an N-terminal DNA binding domain and C-terminal ligand sensing domain (IPR021586) (orange bar), and TATA binding proteins (TBPs, IPR000814) (gray bar) are compared as ratios of UniProt hits per RNA polymerase alpha subunit (RpoA, IPR000722) to account for differences in genome sequence availability among the phyla. Among the archaea, TrmB homologs with a C-terminal ligand sensing domain were not detected in *Methanopyri*, *Methanococci*, and *Theionarchaea*. Archaea with cysteine-containing OxsR homologs are indicated by black and gray circles (with gray indicating that many archaea within this group do not contain OxsR homologs).

OxsR is member of the TrmB family (Pfam PF01978). Like most archaea, H. volcanii encodes multiple TrmB family proteins with all 13 harboring an N-terminal DNA binding domain (IPR002831) classified to the winged helix-turn-helix (wHTH) superfamily (IPR036388) (Fig. 1A). However, only five of these proteins are fused to a C-terminal ligand sensing domain (IPR021586). The remaining eight proteins have a single DNA binding domain including the smallest member of this group: OxsR (123 aa, 14 kDa). Such single-domain TrmB family proteins are widespread among archaeal phyla yet poorly understood in function, while two-domain TrmB homologs with a C-terminal ligand binding domain are more commonly studied but not as widely distributed (Fig. 1B). Within the TrmB family, OxsR is classified to the archaeal cluster of orthologous genes arCOG02242 (66) and by BLASTP comparison is related to homologs of the *Euryarchaeota*, *Crenarchaeota*, and *Asgard* archaea (Fig. 1B). OxsR does not share close relationship to bacterial members of the TrmB family, which are less common than those of archaea (9). Overall, OxsR is a TrmB family protein that has a single wHTH domain and appears conserved in diverse archaeal phyla.

### OxsR is important for recovery of H. volcanii from hypochlorite stress.

**FIG 2:**
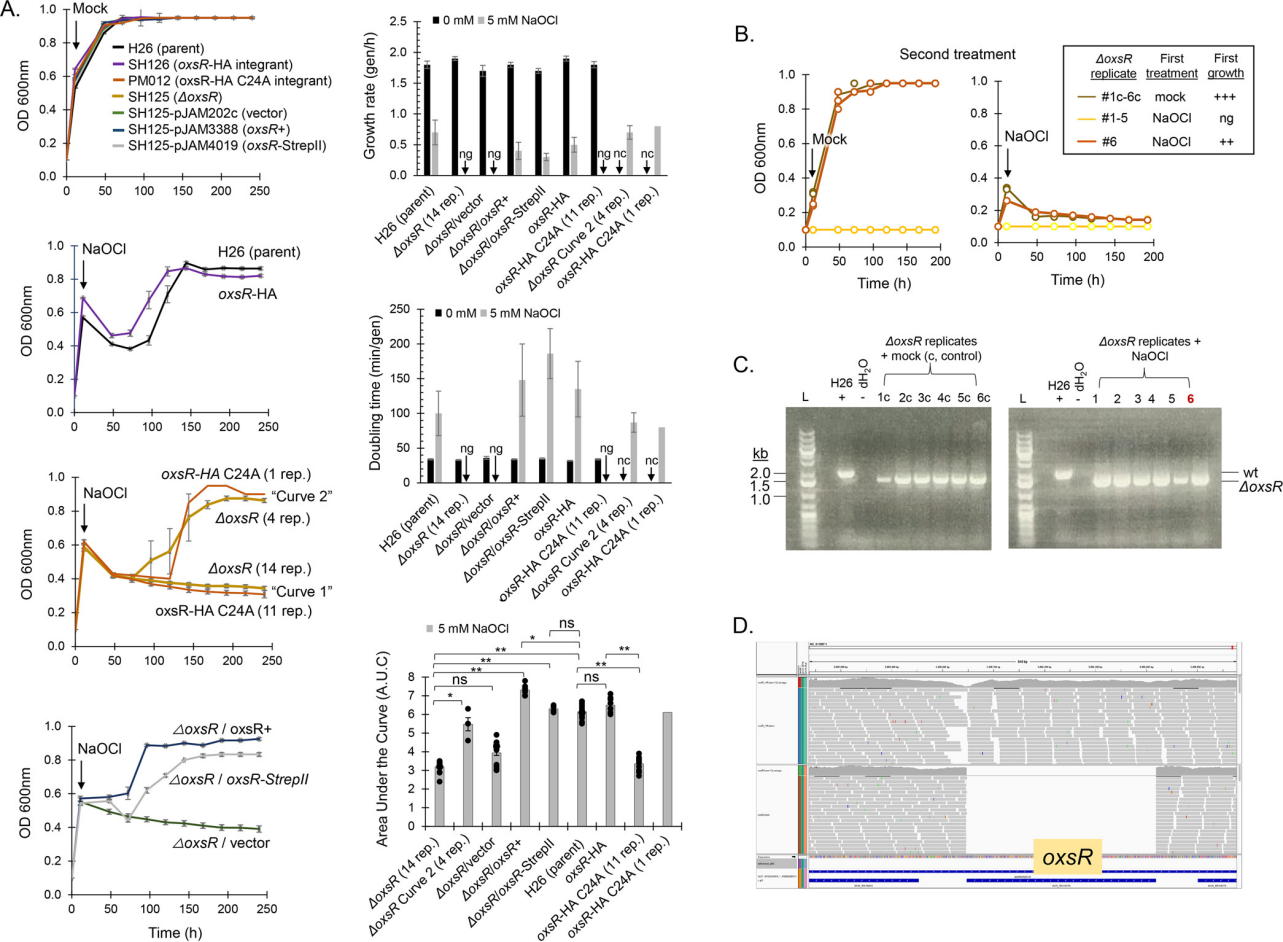
OxsR facilitates recovery of H. volcanii from hypochlorite stress. (A) *S*trains grown in glycerol minimal medium (GMM) to log-phase (11 h, OD600 nm, 0.5 to 0.6) were treated with a mock control or NaOCl (5 mM), as indicated. Growth rates, doubling times, and area under the curve (AUC) analysis based on the trapezoidal rule were determined as indicated. nc, not calculated. ng, no growth detected. Bars represent the average for each factor with error bars representing standard deviation. Significant differences (**, *P*-value ≤ 0.001; *, *P*-value ≤ 0.05) were determined for AUC by the student two-tailed *t* test. ns, not significant. F-test revealed all variances to be equal across the samples where the means were compared. Growth curves were determined based on individual replicates for each strain type: H26, SH125, and SH125-pJAM202c (*n* = 18 total); SH126, PM012, and SH125-pJAM3388 (*n* = 12 total) and SH125-pJAM4019 (*n* = 6 total). Colored curves represent the average growth for the replicates with exception of the *ΔoxsR* mutant and *oxsR*-HA C24A integrant strains treated with 5 mM NaOCl, which are represented by two curves with replicate numbers indicated. Cell growth was monitored at 600 nm (OD600) by direct measurement in the 13 × 100 mm culture tubes using a Spectronic 20+ spectrophotometer (ThermoSpectronic, Filter:600 to 950 nm). OD600 of 0.95 is the maximum value that can be detected by this approach. (B) Analysis of *ΔoxsR* mutant replicates for acquired tolerance to hypochlorite. Three groups of *ΔoxsR* replicates were reexamined for growth and recovery from hypochlorite stress, including: (i) 1c to 6c pooled from the first mock treatment, (ii) 1 to 5 pooled from the first NaOCl treatment that did not display growth, and (iii) replicate 6 that recovered from the first NaOCl treatment. After the first treatment, the samples were diluted to an OD600 of 0.1 in fresh GMM (6 tubes each) and incubated at 42°C with angled rotation for aeration as described in methods. After 10 h, the samples were treated with a mock control or NaOCl (5 mM) as indicated. Growth was monitored at OD600. (C) PCR analysis to assess stability of the *ΔoxsR* mutation. Lanes: L, DNA standard (GeneRuler 1 kb Plus DNA Ladder, Thermo Fisher); *ΔoxsR* mutant (replicate numbers indicated); parent (H26, +); nuclease free water (dH_2_O, –). For PCR, cell culture (5 μL) was mixed with 30 μL of nuclease-free water and boiled for 10 min. Samples were centrifuged for 5 min at 13,000 × *g* and stored at −20°C for 4 days. Tubes were thawed on ice and centrifuged (5 min at 13,000 × *g*) and transferred (1 μL) for use as the template in a 15 μL PCR with primer pair 5/6 (outside the deletion plasmid). (D) Whole-genome resequencing indicates that *oxsR* was deleted from all copies of the genome (no reads were detected). Integrated genomics viewer (IGV) image shows sequencing reads (gray) of H26 parent strain (above) compared with the *ΔoxsR* deletion strain (below). Blue lines in bottom track show the location of the genes in the locus. See Materials and Methods for details.

To examine the biological role of OxsR, the following strains were constructed. The *oxsR* gene was deleted from the H. volcanii H26 (parent) genome using a markerless approach (Fig. S1; Data set S1). The resulting *ΔoxsR* mutant was subsequently transformed with plasmids carrying the *oxsR* gene and the empty vector control. A strain was also generated for pulldown assays by integration on the genome of a hemagglutinin (HA) coding sequence onto the 3′ end of *oxsR*. The *oxsR*-HA integrant strain was further modified to include a C24A mutation as discussed in a later section. An *ΔoxsR* mutant carrying a plasmid for expression of *oxsR* with a C-terminal StrepII tag was also generated to purify OxsR in high salt buffers for biochemical assays. The strains constructed in this manner were subsequently examined for recovery from hypochlorite stress (Fig. 2A). All strains were found to be of comparable growth when cultured under standard conditions in glycerol minimal medium (GMM) (Fig. 2A). By contrast, differences in strain recovery were seen when cells were treated with hypochlorite (Fig. 2A and B). While the parent and *oxsR-HA* integrant were found to fully recover from hypochlorite stress after an 88 ± 8-h lag, the *ΔoxsR* mutant displayed either no growth (curve 1, 14 replicates) or recovered after an extended lag (117 ± 23 h) (curve 2, 4 replicates). The *ΔoxsR* replicates of curve 1 were no longer viable after hypochlorite stress as determined by subculturing to fresh GMM (with no added NaOCl) (Fig. 2B) as well as analysis by plate count in which growth of the mutant was undetectable while the parent and *oxsR-HA* integrant were detected at 1.0 to 1.3 × 10^7^ CFU/mL. The *ΔoxsR* replicates of curve 2 that displayed an extended lag were viable, but did not recover after exposure to a second dose of hypochlorite suggesting the observed phenotype was not due to a suppressor mutation (Fig. 2B). Further analysis by PCR revealed the *ΔoxsR* replicates from curves 1 and 2 retained the markerless deletion of the *oxsR* gene (Fig. 2C), consistent with whole-genome resequencing analysis which demonstrated *oxsR* to be deleted from all genomic copies (Fig. 2D; Data set S1). When performing complementation assays, the *ΔoxsR* mutant was found to be uniformly hypersensitive to hypochlorite in the presence of the empty vector, suggesting that the plasmid posed an extra burden to the cells under these conditions (Fig. 2A). Expression of the *oxsR* gene from this multicopy plasmid with or without a C-terminal StrepII tag facilitated the recovery of the *ΔoxsR* mutant from hypochlorite stress to parental levels (Fig. 2A). This finding suggests that the second site point mutation observed in the *ΔoxsR* genome sequence (noncoding intergenic G > A mutation between *hvo_RS01570* and *hvo_RS01575*; Data set S1) was not the source of the observed hypochlorite recovery defect of *ΔoxsR*. Based on these results, OxsR is important for the recovery of H. volcanii from hypochlorite stress. Furthermore, the minimal effect of the C-terminal HA and StrepII tags on OxsR function during hypochlorite stress indicated these constructs could be used for downstream immunoprecipitation and purification assays.

10.1128/mbio.00633-22.1 FIG S1 H. volcanii mutant strains generated in this study, including SH125, SH126, PM012, PM057 and PM058, and PM059. Download FIG S1, PDF file, 0.1 MB. Copyright © 2022 Mondragon et al. 2022 Mondragon et al. https://creativecommons.org/licenses/by/4.0/ This content is distributed under the terms of the Creative Commons Attribution 4.0 International license.

10.1128/mbio.00633-22.8 DATA SET S1 Data associated with breseq resequencing, ChIP-seq, MEME-MAST, FIMO, and other associated analyses. Download Data Set S1, XLSX file, 1.6 MB. Copyright © 2022 Mondragon et al. 2022 Mondragon et al. https://creativecommons.org/licenses/by/4.0/ This content is distributed under the terms of the Creative Commons Attribution 4.0 International license.

### OxsR binds specific regions on the H. volcanii genome.

**FIG 3:**
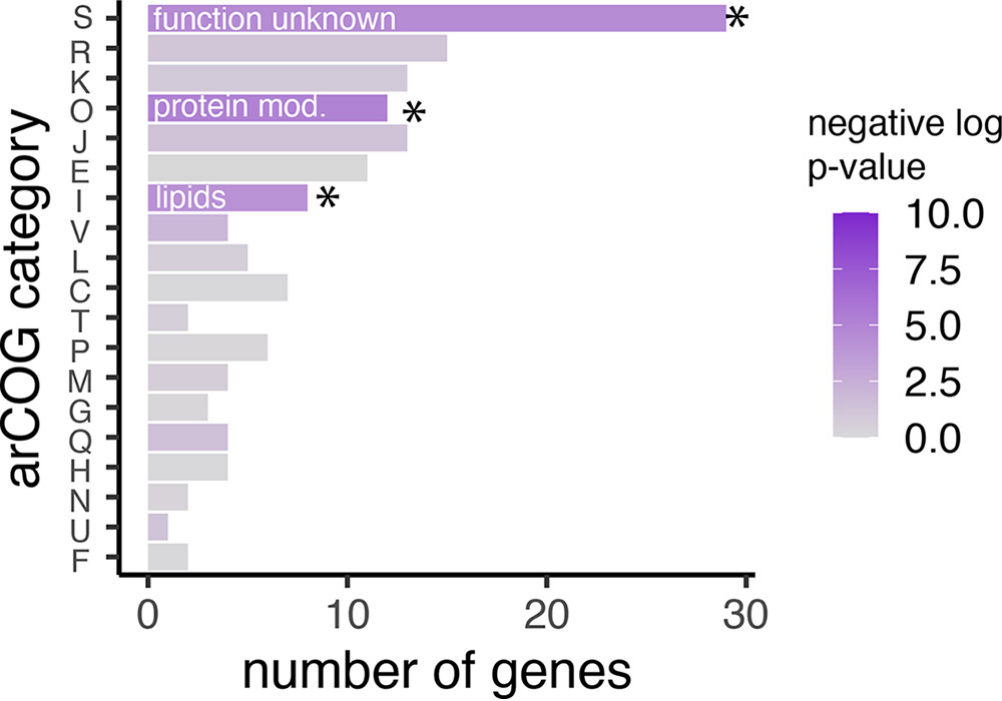
General functional classification of genes predicted to be bound by OxsR according to ChIP-seq data. Gene homologs flanking the intergenic regions of DNA detected to be bound by OxsR through ChIP-seq analysis were clustered by arCOG according to function. Bar lengths represent the number of genes detected in each functional category, and the shading of the bar represents the significance of enrichment in each category (see legend).

OxsR was investigated for its ability to bind specific and/or nonspecific regions of H. volcanii genomic DNA by combining chromatin immunoprecipitation with massively parallel DNA sequencing (ChIP-seq). The parent (H26) and *oxsR-HA* integrant were grown to log-phase in GMM and then treated with hypochlorite or a mock control prior to the ChIP-seq analysis (see Materials and Methods for details). In this approach, OxsR-bound sites were investigated over the chromosome and endogenous plasmids. A total of 29 and 130 sites were found to be putative OxsR interacting regions in the absence and presence of oxidative stress, respectively (Fig. S2, Data set S1). Although many peaks (63%) were located within the coding sequences of genes, OxsR was also found to bind distinct intergenic regions of the genome. Most of the OxsR-bound intergenic sites (49 of 59 total) were detected only in the hypochlorite-treated cells with six operons having two 5′ binding sites. Of the remaining intergenic sites, seven were identified irrespective of the treatment and three were detected only in the absence of hypochlorite (Data set S1). When analyzing the genes adjacent to the OxsR-bound regions by arCOG gene functional analysis (67), nearly half (44%, hypergeometric test *P*-value of enrichment relative to genomic background < 1.1 × 10^−2^) of the encoded proteins clustered to the arCOG [S] group of unknown function, thus, providing limited insight (Fig. 3). However, 12% of the proteins clustered to the arCOG [O] group associated with posttranslational modification, protein turnover, and chaperone functions, including gene homologs associated with thiol relay (e.g., thioredoxin, thioredoxin-like, and disulfide oxidoreductase activity; Data set S1); and 3% to [I] (lipid transport and metabolism, *P* < 6.1 × 10^−5^). Additional functional analysis of the ChIP-seq associated genes by STRING (11.5) (68) corroborated arCOG findings (false discovery rates < 0.0374; Fig. S3). Further inspection of the sites strongly enriched for OxsR binding in intergenic regions (“ChIP-seq high peaks” with height > 1,000-fold enrichment relative to the input control, 27 of 59 total; Data set S1) revealed that at least seven of the linked genes were associated with the synthesis of low molecular weight thiols and thiol relay systems (Table 1). HVO_1043, a member of the DUF1684 family, was included in this thiol relay group, as three-dimensional (3D) modeling and multiple amino acid sequence alignment revealed a conserved Cx_7_C motif with the thiol groups of these cysteines in a proximity typical of function in thiol relay (Fig. S4). Overall, these results reveal OxsR is a TF homolog that binds specific regions of genomic DNA during redox stress. Of the intergenic regions bound by OxsR, an enrichment was observed in the promoter regions of genes associated with protein quality control, thiol relay and other related functions predicted to be important in the recovery from hypochlorite stress.

**TABLE 1:** Gene loci with 5′ regions detected as high peaks by OxsR ChIP seq analysis^*a*^

Gene locus	Protein names	NaOCl (–)	NaOCl (+)	High peak
HVO_1043^*b*^	DUF1684 family protein	V^*c*^	V	V
HVO_A0618^*b*^	Coenzyme A disulfidereductase homolog	V	V	V
HVO_1875	Galactoside O-acetyltransferase (EC 2.3.1.-) homolog	V	V	V
HVO_0198	UPF0213 family protein	V	V	V
HVO_1342^*b*^	Thioredoxin	V	V	V
HVO_0811^*b*^	L-Aspartate decarboxylase (EC 4.1.1.11) homolog		V	V
HVO_0337	Glutaredoxin		V	V
HVO_0040^*b*^	Transmembrane protein		V	V
HVO_0004	NamA family oxidoreductase		V	V
HVO_1031	Thioredoxin-disulfide reductase (EC 1.8.1.9)		V	V
HVO_1166	Chloroplast RNA splicing and ribosome maturation (CRM) domain protein		V	V
HVO_1684	Threonine-tRNA ligase (EC 6.1.1.3)		V	V
HVO_0497	Cold shock protein		V	V
HVO_0251	Glutaredoxin		V	V
HVO_1244	Thioredoxin		V	V
HVO_1668^*b*^	γ-Glutamylcysteine synthetase (EC 6.3.2.2)		V	V
HVO_1522	Hexuronic acid methyltransferase AglP (EC 2.1.1.-)		V	V
HVO_1438^*b*^	YneT family protein		V	V
HVO_1457	Glycoside hydrolase domain protein		V	V
HVO_2157	Uncharacterized protein		V	V
HVO_2859	DUF63 family protein		V	V
HVO_1124	DUF357 family protein			V
HVO_1017	Ketosamine kinase domain protein			V

a Blue highlight, putative function in low molecular weight thiol (LMWT) biosynthesis or thiol relay.

b CG-rich DNA motif CGGnCGnGCG identified 5’ of these gene homologs.

c V, present under the condition.

10.1128/mbio.00633-22.2 FIG S2 Peak loci in chromosome and plasmids by ChIP-seq analysis in the absence (left) or presence (right) of oxidative stress. Download FIG S2, PDF file, 0.02 MB. Copyright © 2022 Mondragon et al. 2022 Mondragon et al. https://creativecommons.org/licenses/by/4.0/ This content is distributed under the terms of the Creative Commons Attribution 4.0 International license.

10.1128/mbio.00633-22.3 FIG S3 Functional enrichment of gene homologs associated with sulfur relay systems by STRING 11.5 analysis. Download FIG S3, PDF file, 0.2 MB. Copyright © 2022 Mondragon et al. 2022 Mondragon et al. https://creativecommons.org/licenses/by/4.0/ This content is distributed under the terms of the Creative Commons Attribution 4.0 International license.

10.1128/mbio.00633-22.4 FIG S4 DUF1684 family protein HVO_1043 and its conserved CX_7_C motif suggest a role in thiol chemistry. Download FIG S4, PDF file, 0.2 MB. Copyright © 2022 Mondragon et al. 2022 Mondragon et al. https://creativecommons.org/licenses/by/4.0/ This content is distributed under the terms of the Creative Commons Attribution 4.0 International license.

### OxsR functions as an apparent transcription factor.

**FIG 4:**
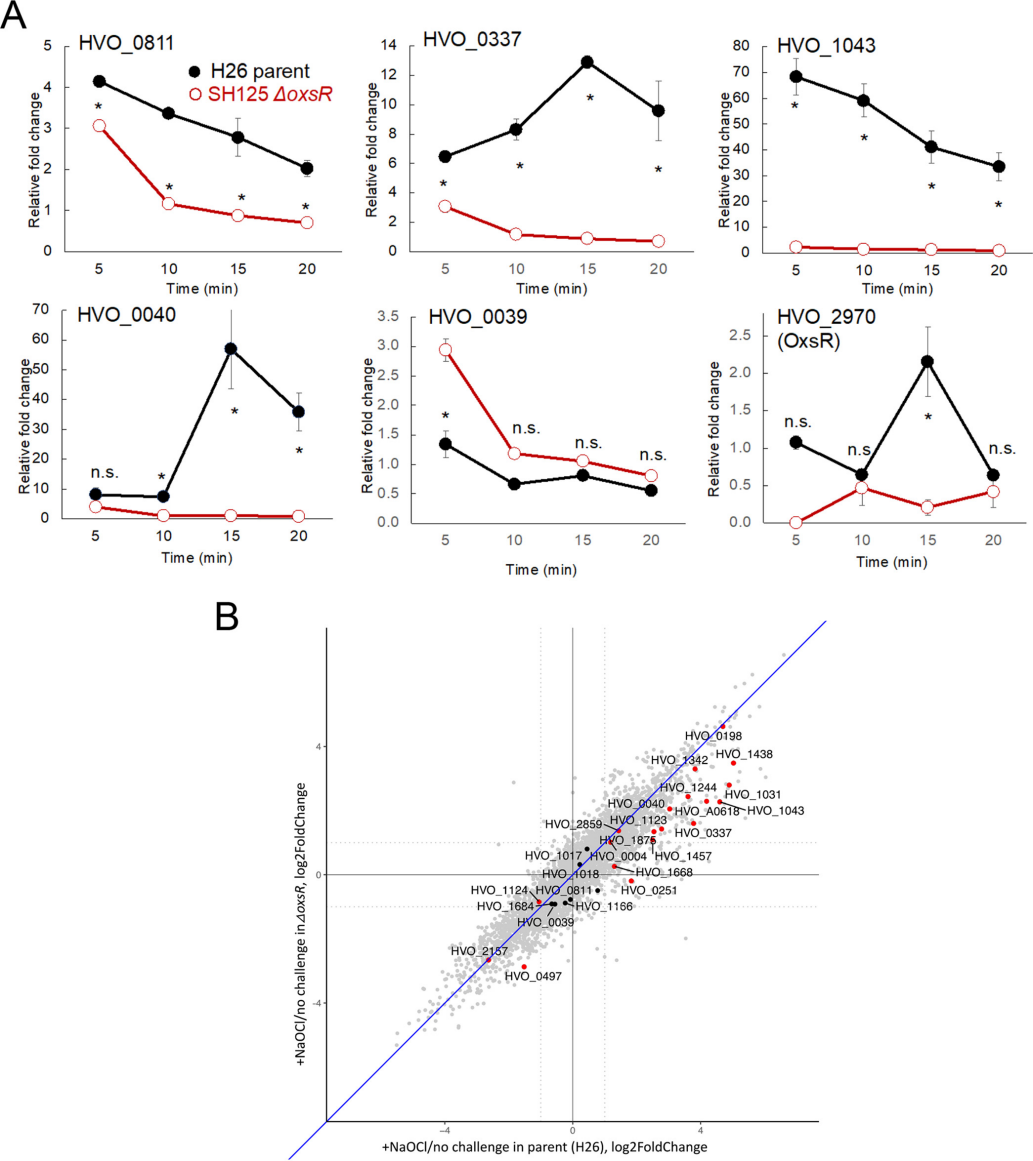
Influence of hypochlorite stress and *oxsR* on the expression of genes identified by ChIP-seq analysis. (A) Expression of select genes identified by ChIP-seq analysis as determined by qRT-PCR after exposure of cells to hypochlorite stress. Relative-fold change represents the transcript abundance ratio of NaOCl: mock treated cells. The H. volcanii H26 parent and SH125 (*ΔoxsR*) mutant cells were grown in GMM to exponential phase and treated with 0 and 2.5 mM NaOCl for 5, 10, 15, and 20 min. Total RNA was extracted and used for quantitative real-time (qRT) PCR. Levels of the gene expression were normalized to the internal reference *ribL* (HVO_1015, 1-fold). Targets of qRT-PCR are indicated as gene locus tag numbers within each panel. *Significant differences between the parent and mutant by the Student’s *t* test analysis (*P*-value ≤ 0.05). n.s., not significant. Data are expressed as mean ± S.E.M. (B) RNAseq analysis highlighting the expression of genes identified by ChIP-seq analysis. Gene expression profile scatterplot comparing the effect of NaOCl (fold change between the presence of 1.5 mM NaOCl and no challenge) on transcript abundance in each strain of parent and *ΔoxsR*, respectively. Gray dots, genes not identified by ChIP-seq; black, genes identified by ChIP-seq, but not significant by RNA-seq; red, genes identified by ChIP-seq and significant by RNA-seq. Gene identifiers are indicated for the genes that are identified by ChIP-seq. See Materials and Methods for details.

Five genes were selected from the ChIP-seq high peak group to determine whether OxsR has an impact on their transcript abundance. In addition, *oxsR* (*hvo_2970*) was included to examine how its transcript levels may correlate with the 3-fold increase in OxsR protein abundance previously identified during hypochlorite stress (65). Transcript levels were monitored by real-time quantitative reverse transcription PCR (RT-qPCR) in the parent and *ΔoxsR* mutant after treatment with hypochlorite or a mock control (*t* = 0) (Fig. 4A). Four of the five genes examined from the ChIP-seq data set were found to be upregulated in the parent after exposure to hypochlorite, including the transcripts of: (i) *hvo_0811* and *hvo_1043*, which displayed a rapid increase; and (ii) *hvo_0040* and *hvo_0337*, which were more delayed in their upregulation. The exception was *hvo_0039*, whose expression remained relatively constant in the parent strain regardless of time or treatment. Hypochlorite stress was also found to stimulate a transient increase in the *oxsR* transcript levels which may in part account for the 3-fold increase in OxsR protein abundance. When comparing the transcript profiles, the *ΔoxsR* mutation was found to impact the transcript abundance of all genes examined. Instead of the increased transcript abundance observed in the parent after treatment, when examining the *ΔoxsR* mutant the *hvo_0040* and *hvo_1043* transcripts were detected at a constitutively low level throughout the time course, while the *hvo_0811* and *hvo_0037* transcripts were only modestly increased in abundance in the early stages of treatment. Counter to the other genes, the *hvo_0039* transcripts were found to be of greater abundance in the early stages of hypochlorite treatment in the *ΔoxsR* mutant compared with the parent. We also investigated the genome-wide gene expression profile with the parent and *ΔoxsR* mutant under the presence/absence of hypochlorite. When the oxidative stress was present, almost half of the genes (1,928 and 1,839) encoded in H. volcanii were differentially expressed in the parent (wt) and *ΔoxsR*, respectively (Data set S1). Next, we examined the expression level of genes identified by ChIP-seq (Table 1) and found that the abundance of these transcripts was reduced by 67% on average across all 27 genes (median 62%) in *ΔoxsR* compared with wt under hypochlorite stress (Data set S1; Fig. 4B). Similar to the qRT-PCR results, the ratio of gene expression of *hvo_0337*, *hvo_1043*, and *hvo_0040* was reduced 42%, 49%, and 68%, respectively. Though upregulated in the parent strain, *hvo_0811* was downregulated in *ΔoxsR* under the oxidative stress condition; however, it is worth noting that the expression change for this particular gene is not statistically significant according to RNA-seq (Data set S1). Overall, both the transcriptomic analyses with RNA-seq and qRT-PCR results suggest that after cells are exposed to hypochlorite, OxsR can act as a transcriptional activator (e.g., *hvo_0040*, *hvo_0337*, *hvo_0811*, and *hvo_1043* regulation) as well as a repressor (e.g., *hvo_0039* regulation). Furthermore, the 3-fold increase in OxsR protein abundance during hypochlorite stress may in part be due to an increase in transcript level.

### Conserved DNA motif in the 5′ region of a subset of genes identified in the OxsR ChIP-seq data set.

**FIG 5:**
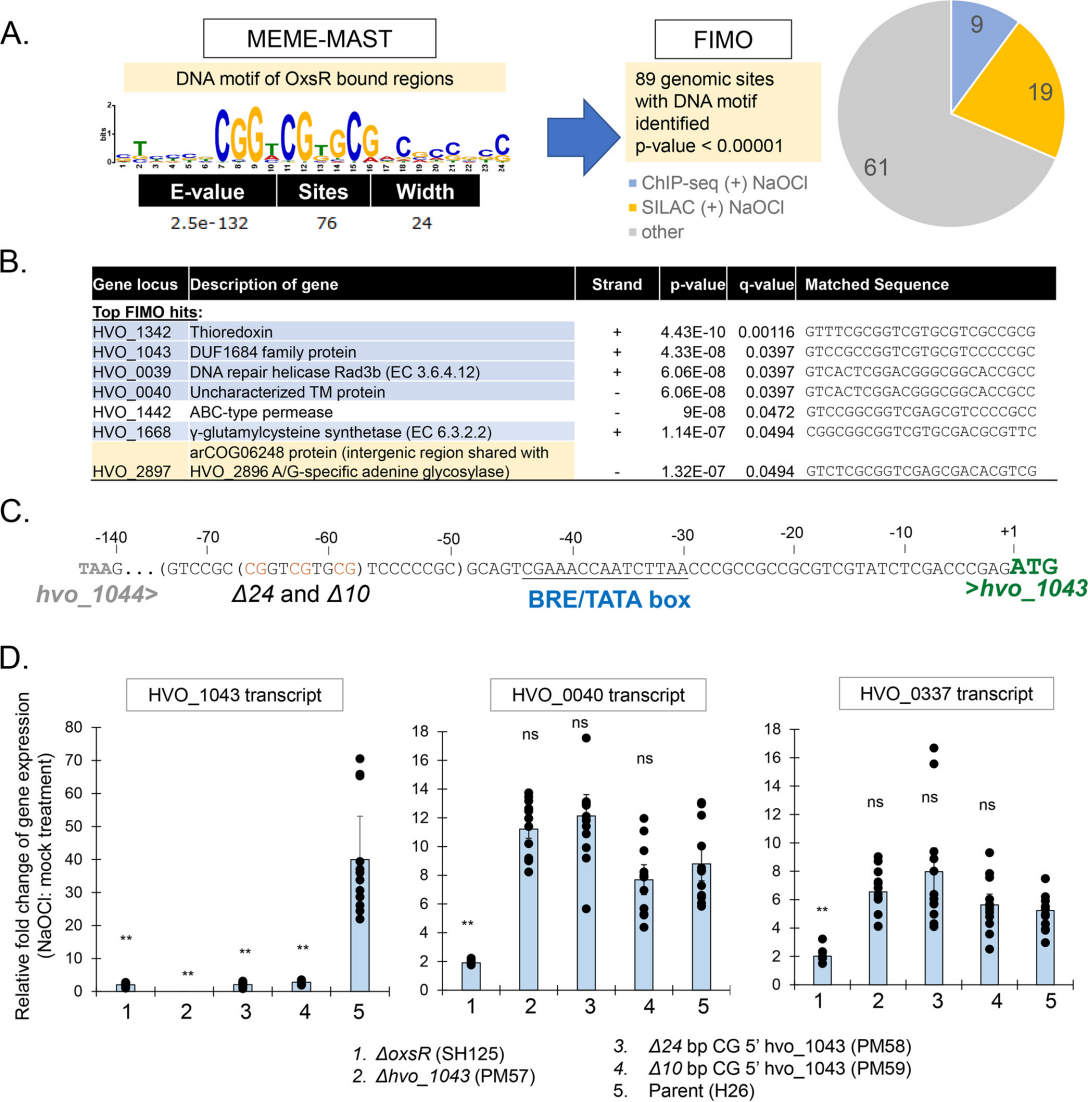
CG-rich DNA motif associated with OxsR-mediated transcriptional activation. (A) CG-rich motif identified to be enriched in OxsR-bound ChIP-seq DNA sequences. The motif was identified by MEME-MAST analysis and was input into FIMO to scan the H. volcanii genome. The 89 sites identified by this approach at a *P*-value < 0.00001 were compared with the OxsR ChIP-seq (blue) and SILAC-based MS (orange) data sets. (B) FIMO hits of the CG-rich motif detected at a *q*-value < 0.05 with the sites common to the ChIP-seq (blue) and SILAC-based MS (orange) data sets highlighted. (C) CG-rich DNA motif upstream of the BRE/TATA box of the *hvo_1043* regulated by OxsR. Parenthesis, CG-rich repeat region targeted for mutagenesis with the CG repeat in orange (Δ24 spans −50 to −74 and Δ10 spans −58 to −67). Underlined, BRE/TATA box region. Green, start codon of the OxsR-regulated *hvo_1043*. Gray, stop codon of *hvo_1044* upstream of *hvo_1043*. D. qRT-PCR analysis of deletions of CG-rich repeat 5′ of *hvo_1043*. H. volcanii strains were grown in GMM to early log phase (OD600 of 0.3 to 0.5) and exposed to 2.5 mM NaOCl or a mock control for 15 min. Transcript levels were monitored in the mutant strains as indicated. The internal reference *hvo_1015* normalized levels of gene expression were at 1-fold relative fold change. Targets of qRT-PCR are indicated for each panel by gene locus tag number. Strains used for preparation of RNA are indicated on the *x* axis. Significant differences between the parent and mutants by the Student’s *t* test analysis (**, *P*-value ≤ 0.01; n.s., not significant) (Exp./Bio: 4; Tech: 3 replicates). See Materials and Methods for details.

*De novo* motif analysis of the DNA sequences of the intergenic regions identified by ChIP-seq was performed. This analysis was executed to identify conserved DNA motifs in the OxsR-bound intergenic sites and to determine whether the location of these motifs relative to the core promoters correlated with expression change. Similar regions from related haloarchaeal genomes were included in the analysis to enhance DNA motif identification. DNA motifs predicted by MEME MAST (69) to be common to the data sets were subsequently used to scan the H. volcanii genome by FIMO (70). FIMO analysis was performed (i) to identify the location of the DNA motifs on the H. volcanii genome; and (ii) to calculate the significance of the findings in terms of false discovery rate (*q*-value) and probability (*P*-value). The FIMO identified sites were then compared with the current ChIP-seq and previously published SILAC data sets, with the latter based on the intergenic regions 5′ of genes encoding proteins of differential abundance after hypochlorite treatment as detected by quantitative SILAC-based MS analysis (65). A semipalindromic DNA motif with evenly spaced CG repeats, CGGnCGnGCG, was identified within OxsR-bound regions (where n and underline represent the bases of low conservation and palindrome, respectively, E-value 2.5 × 10^−132^; Fig. 5A). This motif was detected at 89 sites on the H. volcanii genome at a *P*-value < 0.00001; with about one third of the sites corresponding to 5′ regions associated with the ChIP-seq and SILAC data sets. Of the top seven sites identified at a *q*-value < 0.05, six clustered to at least one of the two data sets (Fig. 5B). Further analysis of the top sites revealed the palindromic DNA motif of CG-repeats was generally positioned 5′ of BRE/TATA box consensus sequences presumed to serve as RNA polymerase binding sites; as depicted for *hvo_1043*, *hvo_1342*, *hvo_1668*, and *hvo_0040* regions (Fig. S5A). The exception was *hvo_0039*, which immediately 5′ of its start codon had a CG-rich region that was not closely related in sequence to the CG repeat but was conserved in diverse *Haloferax* species. This orientation of the CG repeat may explain the repressive function of OxsR on the transcript levels of *hvo_0039*. The CG repeat was further examined for its relationship to the raw sequencing data derived from the ChIP-seq analysis and found to correlate with the highest peak scores for these intergenic regions (Fig. S5B). Thus, our suggestion that this semipalindromic DNA motif is an OxsR-binding site is supported by the ChIP-seq data sets, and its positioning in relationship to basal promoter elements is consistent with the up- or downregulation in transcript levels observed for these genes by qRT-PCR. However, the CG-rich motif did not fully explain all OxsR-dependent activities as (i) not all intergenic regions bound by OxsR have this CG-repeat motif, and (ii) the 12-fold increase observed for *hvo_0337* (glutaredoxin) transcripts during hypochlorite stress requires *oxsR*, yet the gene does not encode the CG-rich motif in its promoter region. Nonetheless, these results suggest for a subset of genes that placement of a CG-rich repeat upstream (e.g., *hvo_0811*, *hvo_0040*, and *hvo_1043*) or downstream (e.g., *hvo_0039*) of the BRE/TATA box promoter consensus element may facilitate the ability of OxsR to activate or repress gene expression in the presence of hypochlorite, respectively.

10.1128/mbio.00633-22.5 FIG S5 A semipalindromic DNA motif and its location in relationship to the promoter consensus sequence element of select gene homologs identified to be OxsR-bound by ChIP-seq analysis. Download FIG S5, PDF file, 0.2 MB. Copyright © 2022 Mondragon et al. 2022 Mondragon et al. https://creativecommons.org/licenses/by/4.0/ This content is distributed under the terms of the Creative Commons Attribution 4.0 International license.

### CG-rich motif required for the OxsR-dependent increase in *hvo_1043* transcript levels after exposure to HOCl.

To determine if the CG-rich DNA motif was important for the observed OxsR-dependent increase in *hvo_1043* transcript abundance during hypochlorite stress, a mutagenesis approach was used. Strains with deletions in the CG-rich motif upstream of the BRE/TATA box consensus sequence of the *hvo_1043* operon were constructed and compared with the H26 parent and *ΔoxsR* mutant by qRT-PCR (Fig. 5C). A *Δhvo_1043* coding sequence mutant was also constructed as a negative control. By this approach, a 40-fold increase in the abundance of *hvo_1043* transcripts was observed after 15 min of hypochlorite treatment in the H26 parent that was not apparent in the *ΔoxsR* or the CG-rich motif mutant strains (Fig. 5C). The *hvo_1043* transcripts were detected only at basal levels in the *ΔoxsR* and CG-motif mutant strains. As expected, *hvo_1043* transcripts were undetectable in the *Δhvo_1043* mutant. As an added control, the transcripts of other genes (*hvo_0040* and *hvo_0337*) were confirmed to be unaffected by the CG-rich motif deletions that were specifically integrated upstream of *hvo_1043*. Based on these results, the CG-rich motif identified upstream of the BRE/TATA box consensus sequence of *hvo_1043* appears important for transcriptional activation of this operon by OxsR during hypochlorite stress. Because of the close sequence similarity of this GC-rich motif upstream of other OxsR-bound operons, we hypothesize that OxsR may bind this motif to regulate expression of at least a subset of genes within its regulon.

### Conserved residues and 3D-structural modeling of OxsR.

**FIG 6:**
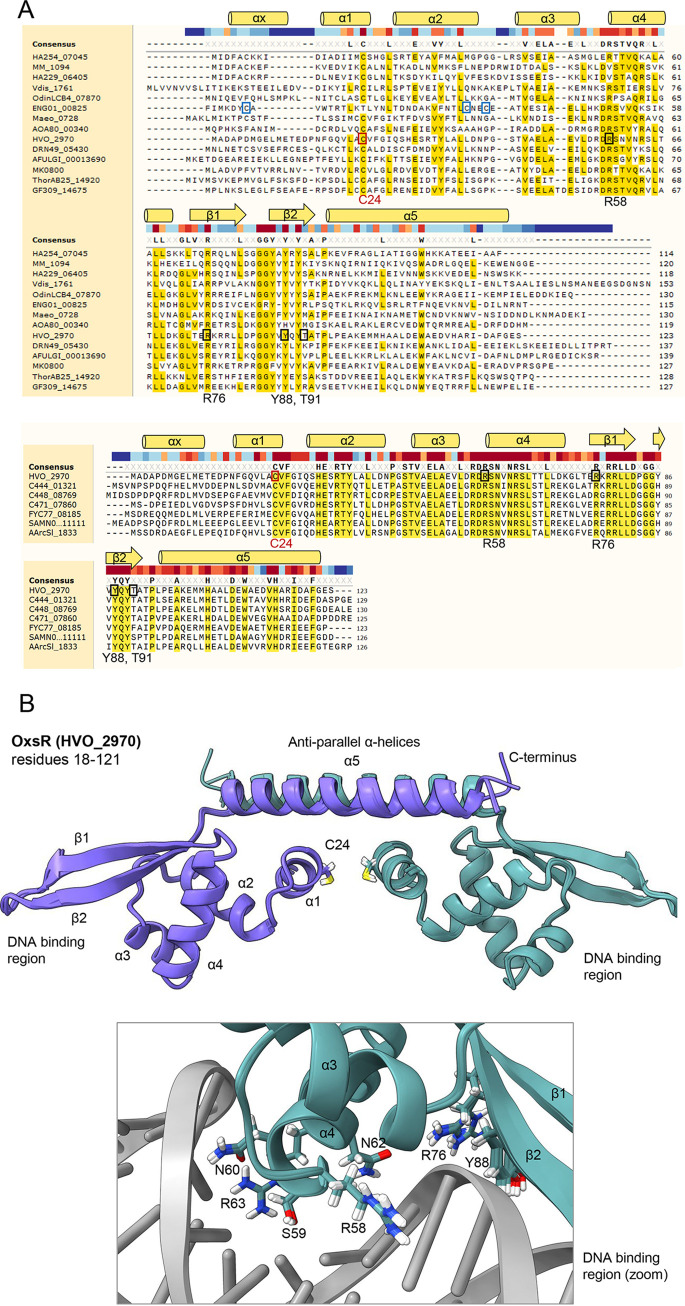
Conserved residues and 3D-structural model of OxsR. (A) Multiple amino acid sequence alignment of OxsR (HVO_2970) to TrmB family proteins. Upper: OxsR aligned to representative homologs from diverse archaea. *Crenarchaeota* (Vdis_1761), *Thaumarchaeota* (DRN49_05430), *Thermoplasmata* (AOA80_00340), *Methanopyri* (MK0800), *Methanomicrobia* (MM_1094), *Methanococci* (Maeo_0728), *Archaeoglobi* (AFULGI_00013690), *Nanoarchaeota* (HA229_06405), *Aenigmarchaeota* (ENG01_00825), *Diapherotrites* (HA254_07045), *Thorarchaeota* (ThorAB25_14920), *Odinarchaeota* (OdinLCB4_07870), and *Lokiarchaeota* (GF309_14675). Lower: OxsR aligned to representative homologs from diverse families of haloarchaea. *Halorubraceae* (C471_07860 and AArcSl_1833), *Natrialbaceae* (FYC77_08185), *Haloarculaceae* (C444_01321), *Halococcaceae* (C448_08769), and *Halobacteriaceae* (SAMN05216226_111111). Residues of OxsR discussed in text indicated by black and red boxes and numbered below the alignment. Blue boxes indicate cysteine residues in the N-terminal region of *Aenigmarchaeota* homolog (ENG01_00825). Predicted α helices and β strands of OxsR indicated above the alignment. Residues at >50% and >95% amino acid sequence identity are indicated by yellow highlighting (upper and lower panels, respectively) with the bar colored dark red to dark blue above the sequence indicating residues of high to low conservation. (B) Three-dimensional-structural model of OxsR (HVO_2970). Ribbon diagram of OxsR in homodimeric configuration (chain A and B in purple and cadet blue, respectively). Residue numbering and secondary structure indicated for chain A. The 3D-structural model generated by RoseTTAfold for residues 18 to 123 is displayed. Arrangement of the 3D model into a homodimer was by comparison to the X-ray crystal structure of the biofilm regulator Sulfolobus acidocaldarius AbfR2 (Saci_1223; PDB: 6CMV). DNA interactions were predicted by comparison to the X-ray crystal structure of the Streptococcus pneumoniae FabT:DNA complex (PDB: 6JBX).

As OxsR was associated with recovery from hypochlorite, which is a potent oxidant, the primary amino acid sequence of OxsR was inspected for conserved residues that may sense oxidant, such as Cys, His and Met (4). Multiple amino acid sequence alignment revealed that a cysteine (corresponding to OxsR C24) was highly conserved in the N-terminal region of OxsR homologs from diverse archaea (Fig. 6A). OxsR was further analyzed by 3D modeling to predict the location of C24 in the protein structure. The 3D structure of OxsR residues 18 to 121 (104 of 123 aa total; 85%) was modeled at >99.8% confidence using the Phyre2 web portal (71). OxsR residues 1 to 123 were also modeled by RoseTTAfold (72). The alignment scores of the two OxsR 3D models were at a mean square deviation (RMSD) of 3.47 Å and template modeling (TM)-score of 0.52, suggesting the models were generally related. The major exceptions were (i) residues 26 to 46 which were structurally undefined in the Phyre2-model, and (ii) residues 1 to 17 which were not modeled by Phyre2 and found in multiple configurations by RoseTTAfold. The highest scoring template by Phyre2 was the X-ray crystal structure of Methanosarcina mazei MM_1094 (PDB: 3R0A), an uncharacterized TrmB family member of arCOG02242, which has two intersubunit disulfide bonds formed between C6 and C17. The single cysteine (C24) of OxsR aligned with C17 of MM_1094. The OxsR quaternary structure was also predicted by comparison to two other high scoring (>99% confidence) templates, including: an assembly of X-ray crystal structures of the *Sulfolobus acdiocaldarius* AbfR2 (Saci_1223) (PDB: 6CMV) (73) and Streptococcus pneumoniae FabT in complex with DNA (PDB: 6JBX) (74). The quaternary model generated by this approach suggested OxsR could form a homodimer linked by an intersubunit disulfide bridge at C24 that would join anti-parallel α helices of the two subunits (Fig. 6B). A separate homodimer interface formed between two anti-parallel α helices at the C-terminus of OxsR was also predicted.

### Cysteine residue (C24) is important for OxsR function.

**FIG 7:**
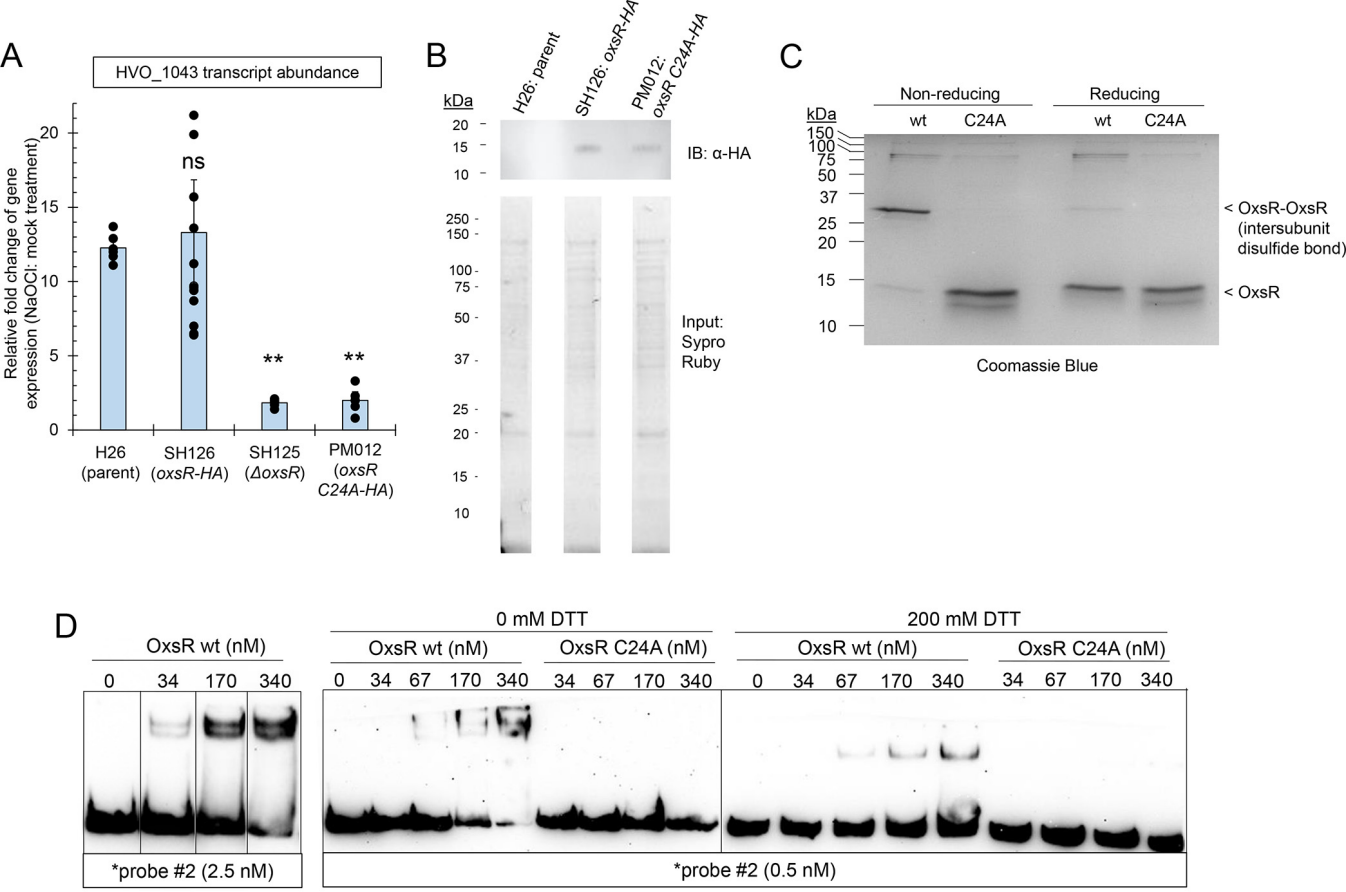
Conserved cysteine residue (C24) and its role in OxsR function. (A) Impact of OxsR C24A on the relative-fold change of *hvo_1043* transcript levels during hypochlorite stress. H. volcanii strains as indicated on the *x* axis were grown to early exponential phase in GMM and treated with 0 and 2.5 mM NaOCl for 15 min. This time frame was found to result in a 10- to 40-fold increased abundance of *hvo_1043* transcripts in the parent strain. Total RNA was extracted and used for qRT-PCR analysis. The internal reference *hvo_1015* normalized levels of the gene expression were at 1-fold relative fold change. Significant differences between the parent and mutant by the Student's *t* test analysis (**, *P*-value ≤ 0.001; *, *P*-value ≤ 0.05). n.s., not significant. (Exp./Bio:2 to 4; Tech: 3 replicates). (B) Detection of OxsR-HA with and without the C24A variant in H. volcanii cells. Strains were grown to early log phase (OD600 of 0.3 to 0.5). Upper panel: Immunoprecipitates (10 μL per lane) were separated by reducing 15% SDS-PAGE for 2 h at 100 V. Proteins were detected by immunoblotting analysis using anti-HA tag HRP (# ab1190) antibodies at 1:20,000 dilution and ECL Prime. Signal was visualized after a 30-s exposure. Molecular mass standards were Precision Plus Protein Kaleidoscope. Lower panel: Total protein input separated by reducing 12% SDS-PAGE prior to immunoprecipitation detected by Sypro Ruby staining is included as control. Samples were normalized as 4 μL (0.04 OD_600_ units cell pellet) per lane. (C) OxsR forms an intersubunit disulfide bond. OxsR wt and C24A proteins were purified with a C-terminal StrepII tag and separated by reducing and nonreducing 16% SDS-PAGE as indicated. (D) Analysis of OxsR binding to DNA by EMSA. Reactions (12 μL) were separated by 9% PAGE in 0.5×TBE buffer pH 8.3 after incubation with 1% formaldehyde for 10 min at room temperature in 50 mM HEPES, pH 7.5, 2 M NaCl, 10% glycerol, 15 mM MgCl_2_, 1 mM EDTA supplemented with 0.25 μg/μL BSA and 0.1 μg/μL sheered salmon sperm DNA. Biotinylated (*) probe was added as indicated. OxsR wt and C24A protein concentrations were based on purified homodimer. See Materials and Methods for details.

To determine whether C24 is important for OxsR function, the H. volcanii genome was modified at the *oxsR* locus to allow for expression of OxsR C24A with a C-terminal HA tag (OxsR-HA C24A). This integrant strain was then examined for recovery of cells from hypochlorite stress and regulation of *hvo_1043* transcript levels. The *oxsR*-HA C24A integrant strain was found to display wild type level growth in the absence of stress but had a severe defect in its recovery from hypochlorite stress (Fig. 2A). Like the *ΔoxsR* mutant, the majority of replicates of the *oxsR*-HA C24A integrant were unable to recover from treatment with hypochlorite. Further analysis of the *oxsR*-HA C24A integrant revealed it was deficient in the upregulation of *hvo_1043* transcript levels during hypochlorite stress (Fig. 7A). While the parent (H26) and the *oxsR*-HA integrant strains showed a robust increase in *hvo_1043* transcript levels when exposed to hypochlorite, the *oxsR*-HA C24A integrant and *ΔoxsR* mutant displayed no such increase (Fig. 7A). To determine whether the C24A had an impact on OxsR protein abundance in the cell, the levels of OxsR-HA and OxsR-HA C24A were compared by anti-HA tag immunoblotting analysis (Fig. 7B). The anti-HA antibodies were found to be specific to the strains expressing the OxsR-HA variants, as no signal was detected in the parent strain devoid of the HA tag. Furthermore, visual inspection of the immunoblots revealed the OxsR-HA C24A and OxsR-HA to be comparable in protein abundance, suggesting the C24A modification did not impact OxsR expression or stability. While amino acid substitution at C24 did not alter OxsR protein abundance, it did eliminate the ability of OxsR to facilitate the recovery of cells from hypochlorite treatment and to upregulate the level of *hvo_1043* transcripts during this stress. Thus, C24 appears to be important for OxsR function as a transcriptional activator when strong oxidants of cellular thiols are introduced into the environment.

### OxsR forms an intersubunit disulfide bridge at C24.

We next examined whether OxsR forms an intersubunit disulfide bridge at C24. The OxsR (wt and C24A) proteins were fused to a C-terminal StrepII tag, expressed, and purified from an H. volcanii *ΔoxsR* mutant. This approach allowed for the synthesis and purification of these proteins in high salt buffers compatible with haloarchaeal protein function and stability (54, 75). The *ΔoxsR* mutant encoding OxsR-StrepII recovered from hypochlorite stress similarly to wild type, revealing the biological activity of OxsR with the StrepII tag was intact (Fig. 2A). Once purified, the OxsR-StrepII (wt and C24A) proteins were analyzed for the formation of disulfide bonds by SDS-PAGE with and without reducing reagent (Fig. 7C). In the presence of the reducing reagent dithiothreitol (DTT), the OxsR wt and C24A proteins migrated at 14 kDa consistent with their theoretical molecular mass. By contrast, when the reducing reagent was excluded from the assay, an additional band of 28 kDa was observed for the OxsR wt protein that was not present in the C24A variant. These results revealed OxsR forms an intersubunit disulfide bridge at C24.

### OxsR binds DNA *in vitro* and C24 appears important for this binding.

Purified OxsR was next examined for its ability to bind DNA carrying the intergenic region with the CG-rich motif 5′ of *hvo_1043* by electrophoresis mobility shift assay (EMSA). The EMSA was modified to allow for analysis of this “salt-loving” protein in buffer supplemented with 2 M NaCl (see Materials and Methods for details). At concentrations ≥ 34 nM, OxsR bound to dsDNA that included the CG-rich repeat of CGGTCGTCCG upstream of *hvo_1043* (Fig. 7D). An approximately 50% reduction in OxsR binding was observed when the CG-rich motif was modified to atGTatTCat on the target DNA (Fig. S6A, B). OxsR binding to the intergenic region 5′ of *hvo_1043* appeared specific based on competition assay (Fig. S6C). The intersubunit disulfide bond was important for the DNA binding of OxsR, as OxsR with the amino acid substitution of C24A did not display DNA binding and inclusion of DTT increased the abundance of 5′-end labeled probe detected in the unbound state and reduced the complexity of gel-shifted bands detected by EMSA (Fig. 7D). Overall, these results reveal OxsR (covalently linked via an intersubunit disulfide bond) can bind dsDNA with sequence that includes the CG-rich motif and corresponds to the intergenic region 5′ of *hvo_1043*. The evidence does not rule out that additional sequence or DNA structural/architectural features are responsible for OxsR binding/recognition, as is seen for MerR family TFs (76).

10.1128/mbio.00633-22.6 FIG S6 Analysis of OxsR binding to DNA by electrophoretic mobility shift assay (EMSA). Download FIG S6, PDF file, 0.6 MB. Copyright © 2022 Mondragon et al. 2022 Mondragon et al. https://creativecommons.org/licenses/by/4.0/ This content is distributed under the terms of the Creative Commons Attribution 4.0 International license.

## DISCUSSION

Here, we advance knowledge of oxidative stress signaling in prokaryotes by associating TrmB-like single winged-helix DNA binding domain proteins from diverse archaea as thiol-based transcriptional regulators of oxidative stress response. TrmB is a large and diverse protein family that accounts for over 10% of the total TFs in archaea and 0.5% of the TFs in bacteria; however, thiol-based sensory mechanisms were not previously reported for this family. Using the TrmB-like OxsR of Haloferax volcanii as a model, we demonstrate that this protein functions as a transcriptional activator and repressor of a large gene coexpression network associated with oxidative stress response. A conserved cysteine residue serves as the thiol-based sensor for this function and likely forms an intersubunit disulfide bond during hypochlorite stress that stabilizes a homodimeric configuration of OxsR with enhanced DNA binding properties.

Our study relies upon H. volcanii cells grown in glycerol minimal medium and exposed to hypochlorite. Thus, the antioxidants present in complex, undefined medium did not complicate our experiments. The hypochlorite used for the environmental cue is a reactive species common to biological systems of high chloride concentration (57). Furthermore, the carbon and energy source, glycerol, was of relevance to hypersaline habitats, where micro algae accumulate large amounts of glycerol for osmotic stabilization, which is released into the environment (77) for use by heterotrophs such as H. volcanii which prefers glycerol over glucose (78).

Evidence suggests OxsR binds a conserved CG-rich DNA motif in promoters of select genes. Promoters with CG-rich motifs located 5′ of the TATA box and BRE consensus were activated by OxsR, whereas those with 3′ CG-rich motifs were repressed. These data suggest that the motif may serve as an OxsR binding site and that the positioning of this site in relation to core promoters (TATA binding protein/transcription factor B binding sites) may determine whether OxsR functions as an activator or repressor (79, 80).

While the CG-rich DNA motif is common to many of the sites bound by OxsR, not all of the OxsR-bound sites share this motif. One possible explanation for this finding is that other protein factors could associate with and influence the type of binding site recognized by OxsR. Consistent with this hypothesis, some gene induction at early time points following exposure to hypochlorite is still observed in the *ΔoxsR* strain for certain OxsR activated genes (*hvo_0811*). Late repression is also possible for other genes (*hvo_0039*), invoking the involvement of another regulator. One candidate is HVO_1360, a small TrmB family protein that shares 28% amino acid identity with OxsR and similarly clusters to arCOG02242 (Fig. S7A). Three-dimensional-structural modeling suggests HVO_1360 forms a wHTH domain flanked on each side by two α-helices much like OxsR (Fig. S7B). Thus, HVO_1360 could potentially form a heterodimer with OxsR that is stabilized by an intersubunit disulfide bond at the anti-parallel α interface of these two subunits (OxsR C24 bound to C21 or C15 of HVO_1360). Formation of this type of complex could alter the DNA sites bound by OxsR and present in the ChIP-seq data set. Hetero- versus homodimerization is found to alter the DNA binding specificity of eukaryotic TFs (81). An alternative explanation is that OxsR has an extensive DNA footprint with limited to no recognizable motif as seen for other TFs (discussed in detail below).

10.1128/mbio.00633-22.7 FIG S7 TrmB family proteins HVO_1360 and OxsR (HVO_2970) are structurally related. Download FIG S7, PDF file, 0.3 MB. Copyright © 2022 Mondragon et al. 2022 Mondragon et al. https://creativecommons.org/licenses/by/4.0/ This content is distributed under the terms of the Creative Commons Attribution 4.0 International license.

TFs with thiol-based redox switches are common in bacteria (4, 5). These TFs are generally classified into 1-Cys and 2-Cys based on sensing through one or two cysteine residues, respectively. Methionine or histidine residues, flavin cofactors, iron, iron-sulfur clusters, and heme centers are also used in bacterial TFs to sense redox status and can be found as added sensors in the thiol-based TFs. ROS or other redox-active compounds can cause specific modifications that lead to conformational changes of these TFs and result in the loss, gain or alteration of their DNA binding activity. Examples of bacterial TF thiol-based modifications include inter- or intrasubunit disulfide bond formation, S-thiolation (mixed disulfides of proteins and low molecular weight thiols), cysteine phosphorylation, and thiol-S-alkylation. These modifications can lead to transcriptional activation, repression, or derepression depending on the TF. Many bacterial TFs with thiol-based switches are inactivated by redox stress leading to transcriptional derepression or deactivation, e.g., 1-Cys OhrR (11, 12), 2-Cys OhrR (13), SarA/MgrA (14), PerR (15), HypR (16), YodB (17), QsrR (18), MosR (19), SarZ (20). One of the most versatile groups of thiol-based bacterial TFs activated by stress are the OxyR homologs of the LysR family including 2-Cys and 1-Cys type. In the presence of peroxide, nitric oxide (NO) or oxidized glutathione (GSSG), OxyR forms intramolecular disulfide bonds (2-Cys OxyR) or other posttranslational thiol modifications that can transform the TF into a transcriptional activator (21) or repressor (22) or lead to derepression (23). In the oxidized state, OxyR forms tetramers that bind DNA with an extensive footprint (~50 bp) composed of repeating spaced elements of limited sequence similarity (82, 83). The well-known bacterial SoxRS operon is also activated by oxidative stress, with its DNA binding activity triggered by the oxidation or nitrosylation of [2Fe-2S] clusters (24, 25). By contrast, bacterial FNR homologs require the presence of an intact 4Fe-4S cluster (otherwise disrupted by oxygen) for function as global transcription regulator when oxygen becomes scarce (26).

Thiol-based redox switch TFs are also found in archaea and eukaryotes. Prior to our work, thiol-based redox switches were identified in TFs of archaea but limited to the ArsR family of transcriptional repressors MsvR (31–33) and SurR (27–30). Upon shifts to oxidizing conditions, these 2- and 5-Cys TFs form intra- and intersubunit disulfide bonds (SurR [29] and MsvR [32], respectively) that result in TF inactivation and transcriptional deactivation and/or derepression. In yeast, Yap1 is identified as a basic leucine zipper (bZIP) TF that uses a thiol-based redox switch to function as a central regulator of oxidative stress response pathways (84, 85). In the presence of H_2_O_2_, Yap1 forms intramolecular disulfide bonds that alter its conformation and mask its nuclear export signal. These changes promote the accumulation of Yap1 in the nucleus and stimulate transcriptional activation of its regulon (86), with formation of an intermolecular thiol intermediate between Yap1 and the thiol peroxidase Gpx3 inhibiting this activity (87). Thus, of the various domains of life, thiol-based TFs that are activated by oxidative stress and mediate a global response have yet to be reported in archaea.

Small wHTH domain proteins that cluster to arCOG02242 similarly to OxsR are characterized in *Crenarchaeota* including Lsr14, Smj12, and others (Table S1). Of these proteins, Lsr14 purifies as a homodimer and forms large footprints (>50 bp) over its own promoter and the alcohol dehydrogenase (*adh*) promoter suggesting it functions as a transcriptional repressor (88–90). The size of the footprints suggests that Lsr14 assembles from a homodimer into higher-order complexes in the promoter region (90). Lsr14 is also found to associate with other DNA binding proteins, such as the benzaldehyde-activated TF (Bald) and the chromatin remodeling proteins Sso7d and Sso10b (Alba), suggesting additional levels of regulation (90). While of low protein abundance in the cell, the related Smj12 displays activities which suggest it has a role in chromatin remodeling including binding DNA nonspecifically, stabilizing the DNA double helix, and introducing positive supercoiling in DNA (91). More recently, Lrs14, AbfR1, and AbfR2 proteins of this arCOG group are correlated with biofilm formation, adhesion, and motility (92, 93), with phosphorylation of AbfR1 Y84 and S87 found important for its binding to promoter regions (94).

10.1128/mbio.00633-22.9 TABLE S1 Representative TrmB family (PF01978) proteins of arCOG02242. Download Table S1, PDF file, 0.2 MB. Copyright © 2022 Mondragon et al. 2022 Mondragon et al. https://creativecommons.org/licenses/by/4.0/ This content is distributed under the terms of the Creative Commons Attribution 4.0 International license.

Based on this study, we find OxsR to have common and distinct properties with the arCOG02242 group representatives that have been characterized. While a CG-rich motif appears important for the DNA binding and transcriptional activity of OxsR at promoter regions, the ChIP-seq enrichment peaks for OxsR were on average 700 bp (Data set S1), which is wider than the peaks observed for other previously characterized halophilic TFs (40, 95). Furthermore, our ChIP-seq analysis revealed six operons to have multiple 5′ intergenic sites bound to OxsR (i.e., *hvo_A0618*, *hvo_2758*, *hvo_1875*, *hvo_0198*, *hvo_1043*, and *hvo_1342*). These results suggest OxsR homodimers may form higher order structures and bind DNA with large footprints similarly to Lsr14. OxsR does not appear to be a highly abundant protein that nonspecifically binds DNA, as specific sites were identified by ChIP-seq analysis, and OxsR-HA could not be detected by Western blotting without prior enrichment by immunoprecipitation, which contrasts with the HA-tagged TF GlpR (96). Furthermore, unlike abundant proteins such as proteasomes, OxsR is detected in only four of the six whole proteome data sets reported in the Archaeal Proteome Project (ArcPP) (97). The most important distinction of OxsR with the previously characterized arCOG02242 members is that it is found to use a thiol-based sensor in the transcriptional response associated with recovery from hypochlorite stress.

Posttranslational modification (PTM) appears important in regulating the activity of the arCOG02242 TFs. We find most members of this arCOG group have conserved cysteine residues located at predicted homodimer interfaces formed by either the antiparallel α1 and/or α5 helices (Fig. 6; Table S1). These cysteine residues form intersubunit and intrasubunit disulfide bonds in the X-ray crystal structures of Sto12a and MM_1094 and, thus, could generally serve as redox sensors that influence TF homodimer stability. Ser/Thr and Tyr phosphorylation provides an additional type of posttranslational regulation to consider in the signaling pathway of TFs of the TrmB family arCOG02242 group (94). While the Y84 and S87 phosphosites of AbfR1 are less conserved among the OxsR homologs than the OxsR C24, we find OxsR to have residues (Y88 and T91) analogous to the sites of AbfR1 phosphorylation suggesting PTM cross talk between a thiol-switch and phosphorylation.

**FIG 8:**
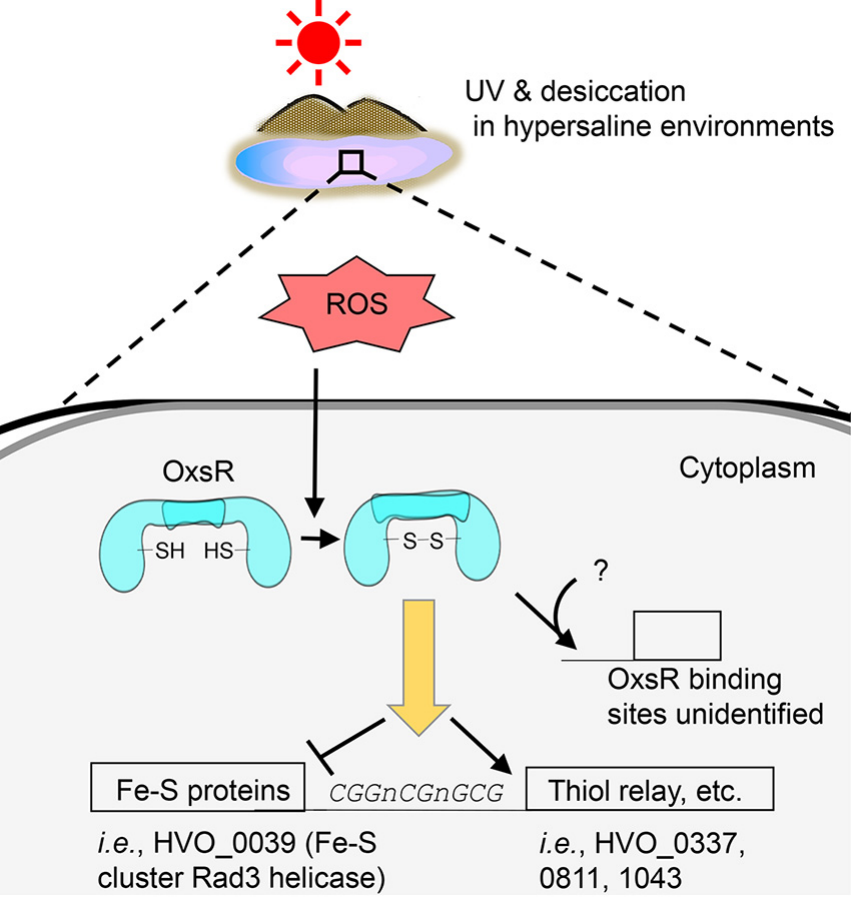
A proposed model of OxsR-mediated oxidative response in H. volcanii. The diagram shows that ROS generated from (a)biotic reactions is sensed by OxsR and then an intersubunit disulfide bond is formed at the conserved cysteine residue, followed by regulating gene expression (activation/repression and target DNA binding).

In this work, we revealed the requirement of OxsR when H. volcanii is exposed to oxidative stress. This work nicely complements previous findings of RosR function in the haloarchaeal oxidative stress response (61–63), as the mode of DNA binding and mechanism of sensing oxidant appear quite distinct between OxsR and RosR (the latter yet to be determined). OxsR binding regions that were mapped by using a genome-wide ChIP-seq approach provide the biological roles not only as an activator for genes involved in amino acid and thiol transfer but a repressor for DNA repair system (Fig. 8). The conserved DNA motif (CGGnCGnGCG) and cysteine residue are mainly contributed to the OxsR binding; however, other factors that interact with OxsR to find/bind to target DNA regions would be remained to be discovered. Overall, this work supports an emerging principle that OxsR which is widespread in most archaeal phyla plays a pivotal role against oxidative stress.

## MATERIALS AND METHODS

### Materials.

Biochemicals and analytical-grade inorganic chemicals were purchased from Fisher Scientific (Atlanta, GA), Bio-Rad (Hercules, CA), and Sigma-Aldrich (St. Louis, MO). Desalted oligonucleotides were from Integrated DNA Technologies (Coralville, IA). DNA polymerases and restriction enzymes were from New England Biolabs (Ipswich, MA) and Clontech Laboratories, Inc. (Mountain View, CA). Hi-Lo DNA standards were from Minnesota Molecular, Inc. (Minneapolis, MN).

### Strains, media, and growth conditions.

Details of strains, plasmids, and primer sequences used in this study are listed in Table S2. Escherichia coli cultures were grown at 37°C in Luria-Bertani (LB) medium. H. volcanii strains were grown at 42°C in ATCC974 complex medium or GMM as previously described (78). Liquid cultures were grown with rotary agitation at 200 rpm. Media was supplemented with 1.5% agar for plates and with novobiocin (Nv, 0.2 μg mL^−1^) or ampicillin (Ap, 100 μg mL^−1^) as needed. Manipulation of H. volcanii strains and DNA was as described by the *Halohandbook* (http://www.haloarchaea.com/resources/halohandbook/). Plasmids were generated in E. coli TOP10 and then transferred to E. coli GM2163 prior to transformation of H. volcanii. Strain and plasmid fidelity was confirmed by Sanger DNA sequencing (Eton Bioscience, Inc., San Diego, CA).

10.1128/mbio.00633-22.10 TABLE S2 List of strains, plasmids, and primers used in this study. Download Table S2, PDF file, 0.2 MB. Copyright © 2022 Mondragon et al. 2022 Mondragon et al. https://creativecommons.org/licenses/by/4.0/ This content is distributed under the terms of the Creative Commons Attribution 4.0 International license.

### Generation of H. volcanii mutant strains.

The H. volcanii *ΔoxsR* mutant (SH125) was generated using H. volcanii H26 as the parent by a *pyrE2*-based pop-in/pop-out deletion method (48). The predeletion plasmid pJAM3380 was generated by ligation of a DNA fragment carrying *oxsR* and 5′ and 3′ flanking regions (about 600 bp each) into the HindIII to XbaI sites of plasmid pTA131. The *oxsR* region carried on pJAM3380 was generated by PCR using primer pair 1/2 and H. volcanii H26 genomic DNA as a template. Deletion plasmid pJAM3381 was constructed by inverse PCR using primer pair 3/4 and plasmid pJAM3380 as a template. Primer pairs used to screen for the SH125 mutant included 1/2, 3/4, and 5/6 (primers outside the deletion plasmid). Plasmid pJAM3388 was created by ligating a DNA fragment carrying *oxsR* into the NdeI and KpnI sites of pJAM809. The *oxsR* region carried on pJAM3388 was generated by PCR using primer pair 5/6 and H. volcanii H26 genomic DNA as a template. This plasmid and the empty vector (pJAM202c) were transformed into the *ΔoxsR* mutant (SH125) for complementation assay. A similar pop-in/pop-out strategy was used to generate the other H. volcanii strains, including SH126, PM057, PM058, PM059, and PM012. Plasmid pJAM3389 used to integrate the *oxsR::HA* coding sequence into the *oxsR* locus was generated by inverse PCR using primer pair 7/8 and plasmid pJAM3380 a template. Strain SH126 was screened using primer pair 9/10. For site-directed mutagenesis to generate pJAM3901 (that encodes OxsR C24A with C-terminal HA tag), plasmid pJAM3389 and overlapping primer pair 11/12 were used to amplify a linear plasmid by PCR that was DpnI treated to remove template and ligated using a KLD Enzyme Mix (NEB). For deletion of *hvo_1043*, the *hvo_1043* region carried on pJAM3919 was generated by PCR using primer pair 27/28 and H. volcanii H26 genomic DNA as a template. Deletion plasmid pJAM3920 was constructed by inverse PCR using primer pair 29/30 and plasmid pJAM3919 as a template. Primer pairs used to screen for mutants included 27/28 (primers outside the deletion plasmid). The deletions of the CG-rich motif 5′ of *hvo_1043* were performed in a similar matter but used different primers. To generate the 24 bp deletion, primer pair 31/32 was used for inverse PCR with pJAM3919 as the template. For the 12 bp deletion of the CG repeat, primer pair 33/34 was used for inverse PCR with pJAM3919 as the template. All constructed plasmids were verified by the Sanger DNA sequencing method (Eton Bioscience, Research Triangle Park, NC). The SH125 (*ΔoxsR*) mutant was also confirmed by comparison to the H26 parent using the breseq pipeline (98) for whole-genome sequencing (Data set S1). Expression plasmids pJAM4019 (OxsR-StrepII) and pJAM4020 (OxsR C24A-StrepII) were generated by ligation of the PCR product generated using the primer pair 5/35 with PM012 genomic DNA and pJAM3901 as the templates, respectively, into the NdeI and KpnI sites of pJAM809.

### Hypochlorite stress growth assay.

For hypochlorite stress growth assays, the following method was used. The H. volcanii strains were streaked from −80°C glycerol stocks onto GMM plates and incubated for 5 days in a closed plastic zippered bag at 42°C. Five isolated colonies were inoculated into 50 mL of GMM in 250 mL Erlenmeyer flasks. Cells were grown to late log phase (OD_600_ of 0.85 to 0.95) at 42°C (200 rpm, rotary shaking). Cells were diluted with fresh GMM to a OD_600_ of 0.1 unit in 95 mL final volume. Aliquots (5 mL) of this cell suspension were transferred into 12 loosely capped 13 × 100 mm culture tubes per strain type and incubated for 10 h at 42°C with aeration using a mini rotator (Glas-Col from Terre Haute in the USA) at a max percent speed setting of 50. Once cells reached log phase (OD600 of 0.4 to 0.6), half of the 12 tubes were randomly selected for supplementation with or without 5 mM NaOCl (sodium hypochlorite reagent grade, available chlorine 10% to 15%, Sigma-Aldrich, #425044-250mL), which forms hypochlorite in solution. The tubes were returned to the mini rotator and cell growth was monitored for 10 days at OD600 using a Spectronic 20+ spectrophotometer (ThermoSpectronic, Filter:600 to 950nm). The experiment was determined to be reproducible (*n* = 12 total; six tubes per strain plus condition for each of two experiments). Initial analysis by circular rotary shaking yielded variable results, most likely due to the microaerobic conditions which promoted extensive incubation times after exposure to hypochlorite.

### Quantitative real-time reverse transcriptase PCR analysis.

H. volcanii strains for quantitative real-time reverse transcriptase PCR (qRT-PCR) analysis were streaked from −80°C glycerol stocks onto defined minimal media (GMM) and incubated for 5 days in a sealed plastic zippered bag at 42°C. Isolated colonies were inoculated into 20 mL of GMM in 125-mL Erlenmeyer flasks. Cells were grown to early log phase (OD_600_ of 0.3 to 0.5) at 42°C (200 rpm). For each strain, aliquots (1 mL) of the cell culture (20 mL) were transferred to 1.5 mL microcentrifuge tubes (RNase-free), and each sample was exposed to 2.5 mM NaOCl for different times (5, 10, 15, and 20 min) at 42°C (200 rpm). A mock control was included for comparison of each time point. Total RNA was isolated from cells using TRI Reagent (#T9424) according to the supplier (Sigma-Aldrich). TURBO DNA-free Kit (AM1907) was used to remove contaminating DNA from the RNA samples according to the supplier’s recommendations (Invitrogen). Only RNA samples with DNA below the limit of PCR detection were further processed. RNA integrity was confirmed by mixing samples 1:1 in 2X RNA loading dye (B0363S; New England Biolabs) and separating by 0.8% (wt/vol) agarose gel electrophoresis in 1X TBE. Only RNA (2 ng) samples with no apparent degradation served as the template for qRT-PCR in 20-μL reactions. The Luna Universal One-Step RT-qPCR kit (E3005L) was used for qRT-PCR analysis following the protocol described by the supplier (NEB) by using a CFX96 real-time C1000 thermal cycler (Bio-Rad). The reverse-transcription was performed under conditions of 55°C for 10 min. The qRT-PCR was performed under conditions of 40 cycles at 95°C for 1 min, 95°C for 10 s, and 56°C for 15 s. An extension of 60°C for 30 s was performed followed by determination of the melting curve under conditions of 95°C for 10 s and increase in temperature from 60°C to 95°C for 5 s each. A single peak revealed by the melting curve indicated a single product. The internal standard HVO_1015 was used to normalize the target mRNA levels, based on finding its transcript levels were unperturbed by HOCl stress. Genomic DNA served as the template to test different primer pairs for PCR efficiency. Primers with PCR efficiency between 95% and 105% are listed in Table S2. For the analysis for qRT-PCR, fold-ratios of relative-fold change in gene expression were calculated using the 2^-ΔΔCt^ method (99). For the statistical analysis, the Student’s *t* test was conducted to compare the mean of gene expression between the H26 parent and *ΔoxsR* mutant strain (SH125). For the qRT-PCR analysis of the *Δhvo_1043* mutant (PM057), strains with deletions of the OxsR binding motif identified 5′ of the promoter region of *hvo_1043* (PM058 and PM059), and the *oxsR*-HA C24A integrant strain (PM012), the same method was used as explained above, except no time course was performed. The strains were exposed to 2.5 mM NaOCl for 15 min, and a mock control was included for each strain. All experiments were performed in biological duplicate or quadlets and technical triplicate.

### Preparation for ChIP-sequencing and data analysis.

Four single colonies of SH126 (*oxsR-HA* integrant) and two colonies of H26 were inoculated in 5-mL GMM and grown aerobically at 42°C to early stationary phase to synchronize growth phase (OD600nm, ~1.0) with shaking (200 rpm). Cells were transferred to fresh 100-mL GMM, and 2.5 mM NaOCl was added for 20 min when cells reached log phase (OD600nm, 0.3 to 0.5) for the oxidative stress group. A mock control was included for comparison. ChIP-seq samples were prepared as the previous method with modifications (100). Briefly, to cross-link, 37% formaldehyde was added to the culture at the final concentration of 1% and the cell culture was incubated on a rocking platform for 20 min at room temperature. A final concentration of 0.125 M glycine was added to stop the cross-linking reaction and the whole cells were washed three times with cold basal salts buffer followed by storage at −80°C until sonication. The cell pellet was thawed and resuspended in 800 μL lysis buffer (50 mM HEPES, 140 mM NaCl, 1 mM EDTA, 1% [vol/vol] Triton X-100, 0.1% [wt/vol] sodium deoxycholate, pH 7.5) containing protease inhibitor cocktail (Thermo Scientific) to shear DNA by Bioruptor 300 sonication system (Diagenode) with 15 cycles of 30 s on and 90 s off at high magnitude. The sheared DNA was monitored to confirm the genomic DNA was a smear between 200 and 800 bp with the highest concentration of fragments ~500 bp by 1.2% (wt/vol) agarose gel electrophoresis. For immunoprecipitation, the sheared DNA was immediately incubated overnight with the complex anti-HA tag antibody (Abcam, ab9110) and Dynabeads protein A (Invitrogen) at 4°C. Enriched DNA/OxsR complexes were eluted by adding 50 μL elution buffer (50 mM Tris, 10 mM EDTA, 1% SDS [wt/vol], pH 8.0) and incubation at 65°C for 10 min. Reverse cross-linking was performed by incubating in TE/SDS (10 mM Tris, 1 mM EDTA, 1% SDS) overnight at 65°C. DNA, of which the RNA was removed, was subsequently extracted by a phenol-chloroform method. Library preparation and deep sequencing were carried out at Duke sequencing core. Sequencing reads were trimmed and controlled their quality (Phred score > 30) by TrimGalore! wrapper pipeline (https://github.com/FelixKrueger/TrimGalore) with default parameters. The preprocessed reads were mapped (alignment rate > 95%) using Bowtie2 (101) to the H. volcanii DS2 reference genome (https://www.ncbi.nlm.nih.gov/genome/1149?genome_assembly_id=170797). The mapped reads were then sorted and indexed by Samtools (102). Peaks (cutoff, Qval < 0.05) were called by MOSAiCS (103) and checked for quality by ChIPQC (104). DiffBind was used to identify significant peaks that were present at least three of four biological replicates (105), and ChIPseeker was used to annotate the peaks (see the peak information in Data set S1) (106). Peak heights reported represent the mean of the ratio of read counts in the IP sample versus input control. Integrative genomics viewer (IGV) was used for the manual evaluation of peak height and peak location, as well as for the data visualization (107). For all gene lists harboring peaks in their upstream coding region, a functional enrichment was performed with arCOG categories (67) based on the hypergeometric distribution test as described previously (108, 109). Code associated with this ChIP-seq analysis are freely available at https://github.com/amyschmid/OxsR_ChIP_WGS.

### RNAseq.

Strains H26 wild-type and mutant (*ΔoxsR*) were streaked on fresh ATCC974 medium plates from −80°C stock and grown for 5 days. Colonies were transferred to fresh ATCC974 liquid media in 13 × 100 mm culture tube and incubated at 42°C for 17 h (aeration by rotary shaking, 200 rpm) to exponential phase (OD600nm of 0.3 to 0.7). Cells were transferred to fresh ATCC974 liquid medium in 13 × 100 mm culture tube and similarly incubated for 15 h until early exponential phase (OD600 nm of 0.3 to 0.5). The culture was transferred to a 125 mL Erlenmeyer flask in 50 mL of ATCC974 liquid medium and similarly incubated for 10 h to reach an early exponential phase. An aliquot (10 mL) of cell culture was transferred to 15 mL conical tubes for each strain and centrifuged for 5 min at 5,000 × *g* at room temperature to remove the complex medium. Cells were washed twice with GMM to remove any remaining ATCC974 medium. The cells were resuspended 50 mL of GMM and divided into two 125 mL Erlenmeyer flasks (25 mL per flask). Cells were exposed to 1.5 mM HOCl or the mock only water for 1 h at 42°C with aeration at 200 rpm. After treatment, 10 mL cultures were transferred to 15-mL conical tubes and harvested by centrifugation (5 min at 5,000 × *g*, 22°C). The supernatant was removed, and the cell pellets were flash frozen on an ice bath with 100% ethanol and NaCl. Cell pellets were stored at −80°C. The RNA was extracted using TRI Reagent (#T9424, Sigma-Aldrich). To verify RNA integrity, the samples were separated by 0.8% (wt/vol) agarose gel electrophoresis (at 90 V for 20 min) in 1X TAE buffer using 2X RNA Loading Dye (#R0641, Thermo Fisher Scientific). The samples were then treated with TURBO DNase-free kit (AM1907, Invitrogen) to remove traces of DNA. Only RNA samples with DNA below the limit of PCR detection were further processed. From each sample preparation, total RNA ≥ 6 μg in 50 μL with RNA Integrity Number (RIN) value ≥ 6 was used to prepare a cDNA library and analyzed by high-throughput sequencing (Novogene) as follows: the abundance of rRNA was reduced using the Ribo-Zero Magnetic kit, and the cDNA was prepared using a 250- to 300-bp insert strand-specific library. The library was sequenced using the Illumina Platform PE150, Q30 ≥ 80% yielding 2G raw data/sample. Sequences from rRNAs were removed from the output, and the remaining sequences were mapped against the H. volcanii DS2 genome with Bowtie2, followed by counting the sequencing reads with HTSeq (110), and finding differentially expressed genes (DEGs) with DESeq2 (111). Two pairwise contrasts were applied: +NaOCl versus mock in parent and +NaOCl versus mock in *ΔoxsR*. For each contrast, DEGs were defined by the fold change cutoff (|log_2-fold_ change| >1) and the adjusted *P* value (padj < 0.05) (Data set S1). Code associated with this analysis is available for download via the repository https://github.com/amyschmid/OxsR_ChIP_WGS.

### Immunoprecipitation of OxsR-HA with and without the C24A mutation.

*Hfx. volcanii* strains H26, SH126 (H26 *oxsR*:HA integrant), and PM012 (H26 *oxsR*:HA C24A integrant) were inoculated from ATCC 974 plates to glycerol minimal medium (GMM) (3 mL in 13 × 100 mm tubes) and grown to late log phase (OD600,0.8 to 1.0). Cells were diluted to an OD600 of 0.03 into fresh GMM (25 mL in 250 mL Erlenmeyer flask) and grown to log phase (OD600, 0.8 to 1.0). Cells were transferred to fresh GMM (200 mL cultures in 1 L Erlenmeyer flask) and grown to stationary phase (OD600, 1.6). Cells were harvested by centrifugation (10,000 × *g* for 30 min, 4°C). Cell pellets were washed with 150 mL 20% salt water (SW, where 20% is composed of 2.46 M NaCl, 88 mM MgCl_2_, 142 mM MgSO_4_, 56.3 mM KCl, 42 mM Tris-Cl, pH 7.5) by centrifugation (10,000 × *g* for 20 min, 4°C). Cell pellets were stored at −80°C for 2 days before use. Cell pellets were resuspended in 0.8 mL of 0.2% (wt/vol) SDS in lysis buffer composed of 50 mM HEPES, 2 mM EDTA, 150 mM NaCl, 1% (vol/vol) Triton X-100, 0.1% (wt/vol) sodium deoxycholate, pH 8.0. Throughout the immunoprecipitation experiment, the buffers were maintained on ice and supplemented with protease inhibitor cocktail according to supplier (Sigma). The resuspended cells were incubated on ice for 15 min and subsequently sonicated with an aspiration probe for 10 cycles (10 pulses, 0.5 s on, 0.5 s off at 30% amplitude) (Sonic Dismembrator Model 500 fitted with a Branson model 102C aspiration probe, Fisher Scientific and Branson Ultrasonics, Danbury, CT) with ice-slurry incubations of at least 1 min between cycles. The sonicated samples were centrifuged (16,873 × *g* for 30 min, 4°C). The supernatant was transferred to a 15-mL Falcon tube (Fisher Scientific), supplemented with 1 μg of α-HA-antibody (ChIP Grade, product # ab9110, Abcam, Cambridge, MA) in 3-mL ice-cold ChIP lysis buffer and incubated for 4 h at 4°C with rocking. During this time, Protein A Dynabeads (50 μL, Invitrogen) were prewashed two times in a 2 mL microcentrifuge tube (natural color) with 1 mL 1 × phosphate-buffered saline (PBS) at pH 7.4 (containing 8.0 g NaCl, 0.2 g KCl, 1.44 g Na_2_HPO_4_, and 0.24 g KH_2_PO_4_ per L) (where the beads were washed by application of slurry to a magnet and removal of supernatant by aspiration). Beads were blocked by addition of 400 μL BSA in PBS buffer (1 mg·mL^−1^). The BSA-bead slurry was added to the preincubated sample and further incubated overnight (rocking at 4°C). After incubation, the supernatant was removed from the beads (via magnet and aspiration), and the beads were resuspended in ice-cold lysis buffer (1 mL). The bead-slurry was transferred to a fresh 2-mL tube, and the supernatant was removed by application to a magnet and aspiration. This wash step was repeated for a total of two times and followed by subsequent washing steps that were each repeated twice with the following buffers: wash buffer 1 (lysis buffer with 150 mM NaCl), wash buffer 2 (10 mM Tris-Cl, 2 mM EDTA, 25 mM LiCl, 1% [wt/vol] Nonidet P-40, 1% [wt/vol] sodium deoxycholate, pH 8.0) and TE buffer (10 mM Tris-Cl, 1 mM EDTA, pH 8.0), respectively. After washing, the beads were resuspended in 100 μL 2 × SDS reducing buffer (125 mM Tris-HCl, pH 6.8, 20% glycerol, 4% SDS, 0.1% bromophenol blue and 5% β-mercaptoethanol) and boiled for 10 min. The samples were centrifuged (5,000 × *g* for 5 min, room temperature). The supernatant was stored in a fresh 2-mL microcentrifuge tube and analyzed by immunoblotting. The remaining supernatant was stored at −20°C for future use.

### Immunoblotting (western) analysis.

Immunoprecipitants (10 μL per lane) were separated by reducing 15% SDS-PAGE. Proteins were transferred to PVDF membrane at 4°C for 14.5 h at 30 V using the mini transblot module in transblot buffer (10 mM MES buffer pH 6 and 10% [vol/vol] methanol) according to the supplier’s instructions (Bio-Rad). The membrane was removed from the cassette, and the location of the gel and protein standards were marked on the membrane using a pencil. The membrane was placed upright in an 18 cm × 10 cm plastic container and rinsed with 30 mL of 1x TBST (50 mM Tris-Cl, pH 7.5, 150 mM NaCl, and 0.5 mL/L Tween 20) for 30 min. The membrane was soaked with 100% methanol and dried for 1 h at room temperature under laminar airflow. The membrane was reactivated with 100% methanol, washed briefly with 1X TBST three times, and blocked for 3 h at 4°C in 60 mL blocking buffer composed of TBST buffer supplemented with 5% (wt/vol) BSA (Sigma Life Science). During the blocking stage, the membrane was gently rocked using the Lab-Line Rocker on medium (5 or 6) setting. The blocking solution was replaced with a solution of anti-HA tag horseradish peroxidase (HRP) antibody (ab1190) diluted to 1:20,000 in 60 mL of the blocking buffer. The membrane was incubated with gentle rocking for 1 h at 4°C. The membrane was rinsed for 1.1 h at room temperature with 50 mL TBST buffer seven times using the high setting of the rocker. The HRP signal of the antibody: protein complexes was visualized on the PVDF membrane by chemiluminescence using 2 mL of Amersham ECL Prime from CGE Healthcare Life Sciences and exposure to the iBright FL1500 Imaging System (A44241).

### Purification of OxsR wt and C24A proteins for *in vitro* analysis.

*H volcanii* SH125 (H26 Δ*oxsR*), carrying pJAM4019 and pJAM4020, were used for purification of OxsR wt and C24A proteins with a C-terminal StrepII-tag. Strains were grown to stationary phase (OD600 2.2 to 3.0) at 42°C in ATCC974 rich medium supplemented with novobiocin (0.3 μg/mL) (200 rpm rotary shaking for ~3 days). Cultures (4 × 750 mL in 2.8 L Fernbach flasks) were harvested by centrifugation (1,912 × *g*, 20 to 30 min at 25°C). Cell pellets were stored at −80°C until use. Cell pellets were resuspended in 5 mL to 1 g (wet weight) cells in lysis buffer (100 mM Tris-Cl buffer, pH 7.5, 2.0 M NaCl, 1.0 mM TCEP, 1 mM CaCl2, 3 mM MgCl2, DNase I [10 μg/mL], and Pierce EDTA-Free Protease Inhibitor Tablets [Thermo Fisher Scientific, USA, Cat No. A32965; 1 tablet/50 mL]). Resuspended cells were lysed using the French press method to a minimum high ratio of 140 (2,000 to 2,500 lb/in^2^) (Glen-Mills, NJ, USA). The cell lysate was clarified by centrifugation (13,177 × *g*, 30 min, 4°C) and sequentially filtered using a 0.45-μm PES filter followed by a 0.22-μm PES filter (Corning Inc., NY, USA). Pierce centrifuge columns of 5 mL volume (Thermo Fisher Scientific, USA, Cat. No. 89897) were packed with 1 mL of slurry (0.5 mL bed volume of resin) of Strep-Tactin Superflow Plus Resin (Qiagen, USA, Cat. No. 1057978). Resin was equilibrated twice by adding 10 bed volumes of binding buffer (100 mM Tris-Cl buffer, pH 7.5, 2.0 M NaCl, 1.0 mM TCEP) and centrifuging (500 × *g*, 1 min at 25°C). Clarified lysate (5 mL at 2.7 mg protein/mL) was added to equilibrated resin and incubated at 4°C for 1 h with rotation at 200 rpm using a tube revolver (Thermo Fisher Scientific, USA, Cat No. 88881001). After incubation, sample was centrifuged (500 × *g*, 1 min at 25°C) and flowthrough was collected. Application of clarified lysate, incubation and centrifugation were repeated with 5 additional mL of clarified lysate. Resin was washed twice with 10 bed volumes (5 mL total) of binding buffer before applying 2 bed volumes (1 mL) of elution buffer (100 mM Tris-Cl [pH 7.5), 2.0 M NaCl, 1.0 mM TCEP, 5 mM desthiobiotin). Resin was rotated (200 rpm) at RT for 30 min. Elution fractions were collected by centrifugation (500 × *g*, 1 min at 25°C). Protein concentration was estimated using Quick Start Bradford Protein Assay (Bio-Rad, CA, USA) according to manufacturer’s instructions. Purity was assessed by comparing the proteins of cell lysate, flowthrough and elution fractions separated by reducing 16% SDS-PAGE and staining with Coomassie brilliant blue R-250. After elution, the resin was stripped with 10 bed volumes (5 mL) of dH_2_O followed by 10 bed volumes (5 mL) of 0.5 NaOH and an additional 10 bed volumes (5 mL) of dH_2_O. Column was regenerated by washing 3 times with 5 bed volumes (2.5 mL) of regeneration buffer (100 mM Tris-Cl buffer, pH 7.5, 2.0 M NaCl, 1.0 mM TCEP, 1.0 mM HABA) and washed with binding buffer until red color disappeared from the resin. Resin was stored in 5 mL binding buffer at 4°C.

### Intersubunit disulfide bond assay.

Purified StrepII tagged OxsR (wt and C24A) proteins were dialyzed overnight in 100 mM Tris-Cl buffer, pH 7.5 supplemented with 2 M NaCl. Protein samples were incubated with 0, 10, 100, and 200 mM DTT (25 min) on ice. DTT at 200 mM was found optimal for OxsR reduction and is within the concentration range used for treatment of other thiol-based TFs, such as bacterial OxyR (112). After incubation, samples were diluted 1:1 in SDS-loading buffer, boiled for 10 min, cooled, and separated by 16% SDS-PAGE. Proteins were stained with Coomassie brilliant blue R-250 and imaged using an iBright FL1500 Imaging System (Invitrogen).

### Halophilic electrophoretic mobility shift assay.

DNA probes for the Halophilic electrophoretic mobility shift assay (hEMSAs) were generated by PCR using oligonucleotides, where one of the primers was 5′-end biotinylated for the labeled probes (Table S2). Primer pairs 36/38, 36/39, and 36/40 were used to generate 5′-end biotinylated probes 1, 2, and 3, respectively. Prior to use, the 5′-end-labeled probes were purified by DNA agarose gel electrophoresis using a QIAquick gel extraction kit. Primer pairs 37/38 and 41/42 were used to generate uniform preparations of probe 1 (179 bp) and probe 4 (248 bp) by PCR, respectively. Prior to use, these unlabeled PCR products were extracted by phenol:chloroform:isoamyl alcohol (25:24:1) and ethanol precipitation (113). Purified OxsR wt and C24A proteins were exchanged into binding buffer (50 mM HEPES, pH 7.5, 2 M NaCl, 10% glycerol, 15 mM MgCl_2_, 1 mM EDTA) immediately prior to use. These OxsR proteins (34 nM to 5 μM) were incubated (12 μL reactions) with the 5′-biotinylated dsDNA PCR products (0.5 to 2.5 nM) in binding buffer supplemented with ultrapure sheared salmon sperm DNA at 0.1 μg/mL (Ambion AM9680) and molecular biology grade BSA at 0.25 μg/mL (New England Biolabs) for 10 min at room temperature with 1% (vol/vol) formaldehyde. Reactions were supplemented with DTT (0 to 200 mM) and “unlabeled” dsDNA probe (0 to 3.3 μM) as indicated. After incubation, the samples were quickly applied without loading dye to 10% PAGE gels in 0.5 × TBE buffer pH 8.3 (110 mM Tris, 90 mM borate, 2.5 mM EDTA) preequilibrated with 0.5x TBE buffer pH 8.3 at 20 mA for 2 h (4°C). Samples were separated by electrophoresis at 4°C for 1.5 h at 60 V followed by 2 to 2.5 h at 120 V. After electrophoresis, DNA and nucleoprotein complexes were transferred to a nylon membrane (BrightStar Plus, Ambion) equilibrated in 0.5x TBE pH 8.3 using the trans blot semidry system (Bio-Rad) at 20 V for 20 min. DNA and nucleoprotein complexes were cross-linked to the membrane via UV radiation using the autocrosslink mode (UV Stratalinker 2400, Stratagene) and were detected using a Chemiluminescent Nucleic Acid Detection Kit (Thermo Scientific) with visualization using an iBright FL1500 Imaging System (Invitrogen).

### Computational prediction of OxsR-binding DNA motifs.

DNA fragments identified by ChIP-seq to be bound to OxsR were tested for common DNA motif(s) using the MEME Suite v. 4.12.0 (69). Sequences were input into the *de novo* motif detection mode of MEME-MAST with the following parameters: any number of repeats, max width of 24 bp, and 3 output motifs. Two DNA sequence sets were used as input. The first set, which did not generate DNA motifs of high significance, included all DNA sequences bound by OxsR. The second set, which identified the CG-repeating DNA motif CGGnCGnGCG (E-value reported in the text represents the expected number of sequences in a random database of the same size that would match the motif), was a subset of the OxsR-bound DNA sequences and was supplemented with analogous intergenic regions from related haloarchaeal genomes. These related regions were retrieved by comparing the deduced protein sequences of the flanking genes by Basic Local Alignment Search Tool using BLASTP (protein-protein BLAST) (114). The DNA sequences 5′ of the genes encoding these homologs were retrieved using the graphics tool within NCBI nucleotide portal (https://www.ncbi.nlm.nih.gov/nuccore/). DNA motifs identified in this manner were compared with shuffled sequences to determine significance. DNA motifs found to be significant (based on uniqueness to the unshuffled sequences) were input into the FIMO algorithm (70) to scan the *Hfx. volcanii* genome database of 4,073 sequences and 1,428,983 residues (using the pull down menu “Upstream Sequences: Prokaryotic” and “Haloferax volcanii DS2 uid46845”). The DNA sequences used to identify the CGGnCGnGCG motif, and the FIMO output of the genome scanning are provided in Data set S1.

### Computational prediction of OxsR 3D structure.

Protein sequences from UniProtKB accession numbers D4GY41 and D4GXQ1 were used to model 3D structures of OxsR and HVO_1360, respectively. The Phyre2 web portal (71) was used to predict structure by fold recognition threading. RoseTTAfold (72) was used for *de novo* structure prediction. The 3D models were visualized and compared using ChimeraX (115).

### Data availability.

The ChIP-seq and RNAseq data discussed in this publication have been deposited in the NCBI Gene Expression Omnibus (116, 117) and are accessible through GEO Series accession number GSE196894 (https://www.ncbi.nlm.nih.gov/geo/query/acc.cgi?acc=GSE196894) and GSE204840 (https://www.ncbi.nlm.nih.gov/geo/query/acc.cgi?acc=GSE204840), respectively. The whole genomic sequence (WGS) data for the *ΔoxsR* mutant (SH125) and *oxsR-HA* integrant (SH126) are deposited with accession number PRJNA806939 in the Sequence Read Archive (SRA) (118). Code associated with the ChIP-seq and RNA-seq data analysis are freely available for download via GitHub: https://github.com/amyschmid/OxsR_ChIP_WGS.
