# Supplementary material for: TrmB Family Transcription Factor as a Thiol-Based Regulator of Oxidative Stress Response
Source: mBio. 2022 Jul 20;13(4):e00633-22. doi: 10.1128/mbio.00633-22 (PMC9426492; doi:10.1128/mbio.00633-22)
Supplement: FIG S1 [file mbio.00633-22-s0004.pdf]

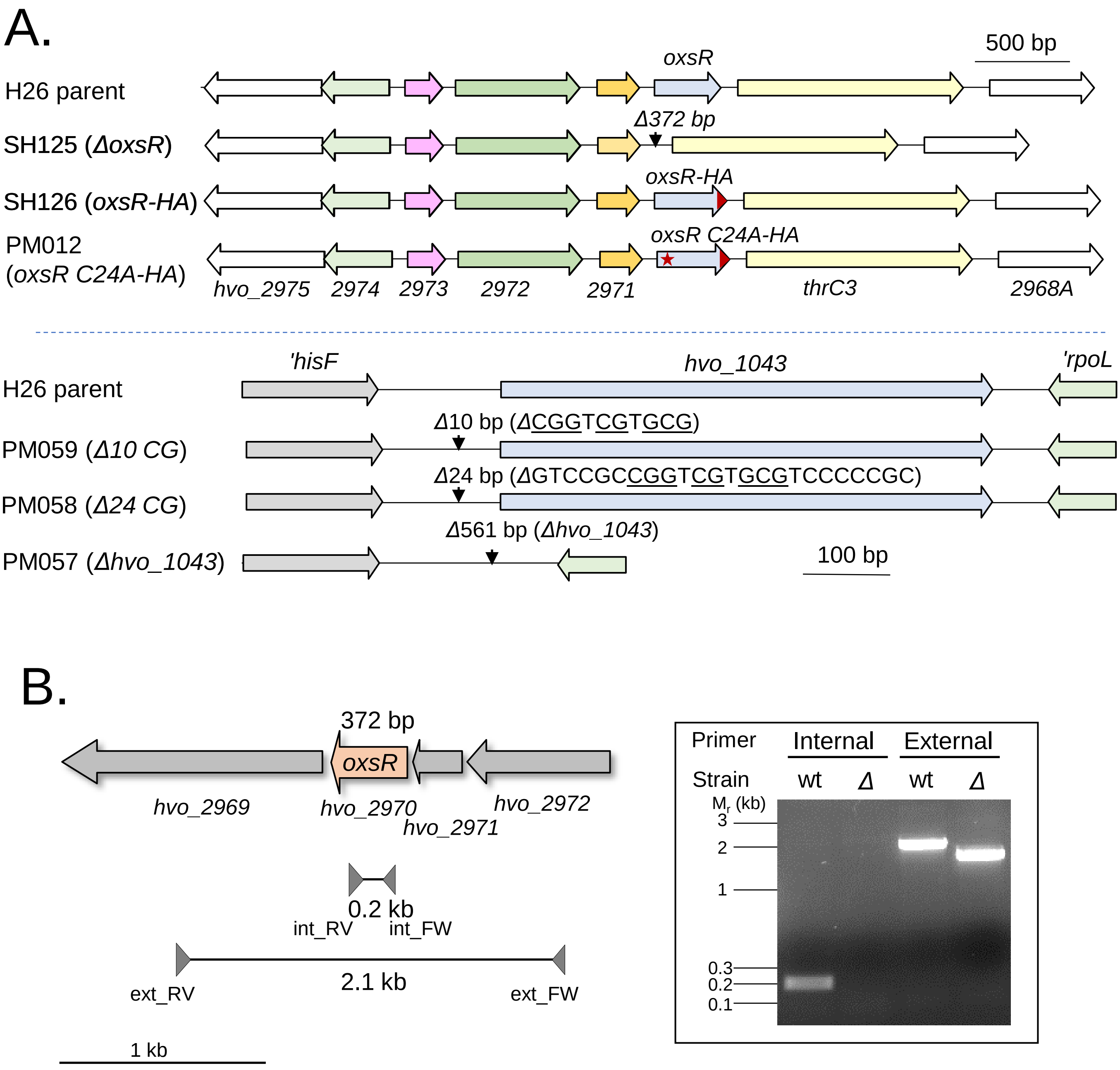

Figure S1. *H. volcanii* mutant strains generated in this study including SH125, SH126, PM012, PM057, PM058, and PM059. A. Genomic neighborhood of the mutant strains. Arrows represent the open reading frames with associated gene locus tag numbers indicated below. Triangle represents the site of the genomic deletion. Bar, scale 500 and 100 bp as indicated. B. Strategy used for markerless deletion as exemplified by generation of SH125. PCR was applied to confirm the *oxsR* gene deletion. A target gene specific primer set (lane 1 and 2) and an external primer set (680 – 970 bp flanking region from the target gene, lane 3 and 4) were used to confirm the *oxsR* gene deletion. Lane 1 and 3, gDNA from wt *Hfx. volcanii*; lane 2 and 4, gDNA from SH125. Bar, scale 1 kb as indicated.
