# Supplementary material for: TrmB Family Transcription Factor as a Thiol-Based Regulator of Oxidative Stress Response
Source: mBio. 2022 Jul 20;13(4):e00633-22. doi: 10.1128/mbio.00633-22 (PMC9426492; doi:10.1128/mbio.00633-22)
Supplement: FIG S2 [file mbio.00633-22-s0005.pdf]

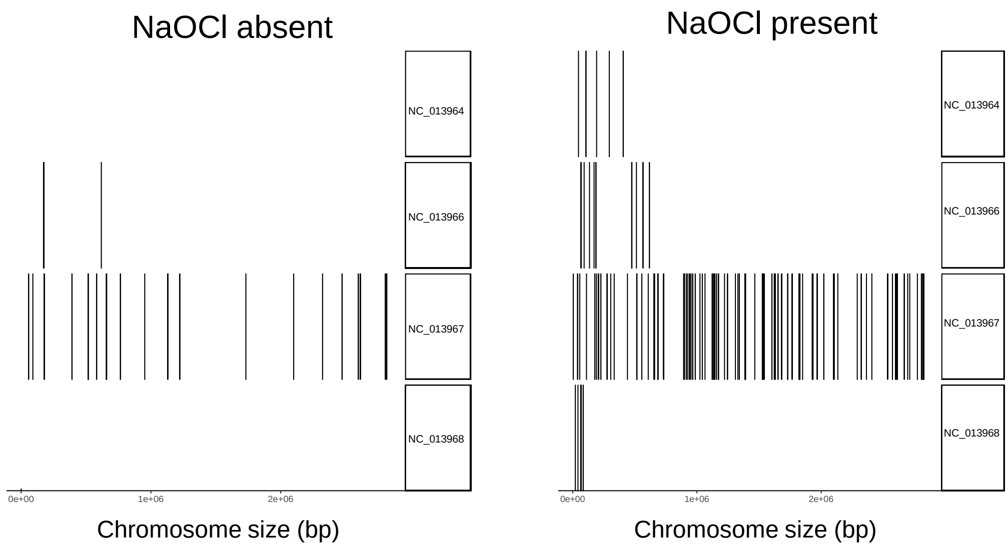

Figure S2. Peak loci in chromosome and plasmids by ChIP-seq analysis in the absence (left) or presence (right) of oxidative stress. NC_013967, main chromosome (2.85 Mb); NC_013968, pHV1 (0.09 Mb); NC_013965, pHV2 (0.01 Mb); NC_013964, pHV3 (0.44 Mb); NC_013966, pHV4 (0.64 Mb).
