# Supplementary material for: TrmB Family Transcription Factor as a Thiol-Based Regulator of Oxidative Stress Response
Source: mBio. 2022 Jul 20;13(4):e00633-22. doi: 10.1128/mbio.00633-22 (PMC9426492; doi:10.1128/mbio.00633-22)
Supplement: FIG S4 [file mbio.00633-22-s0007.pdf]

A.

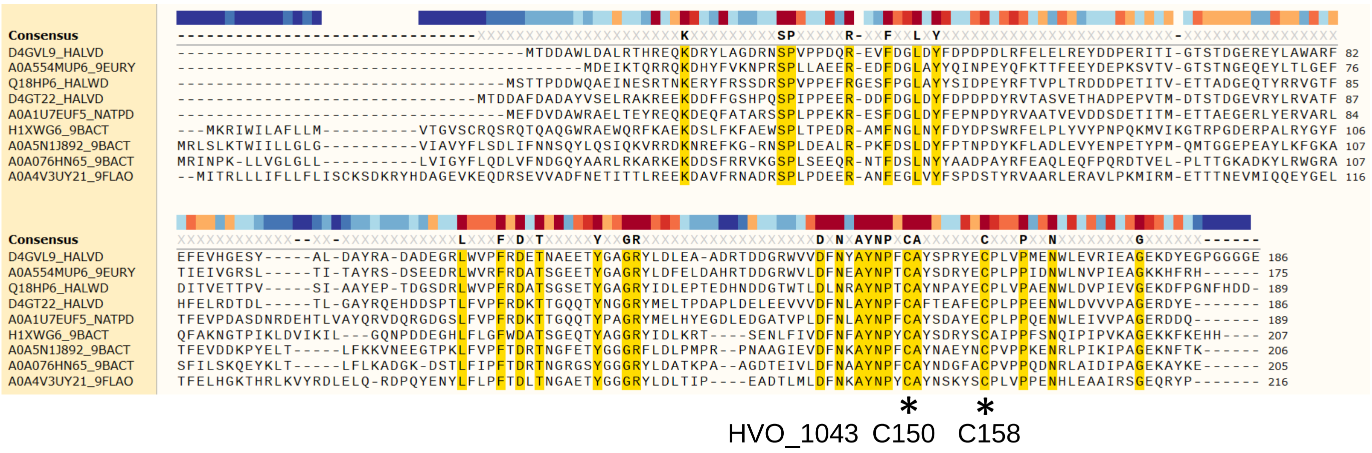

B.

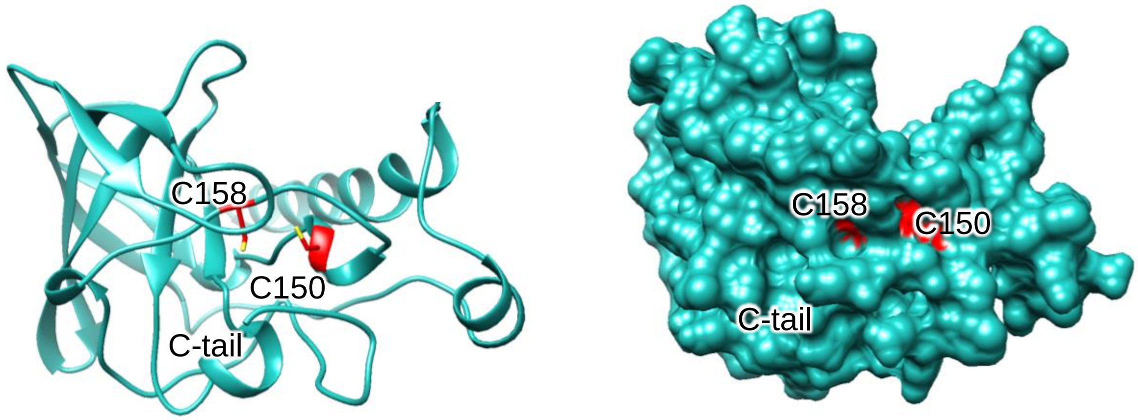

Figure S4. DUF1684 family protein HVO_1043 and its conserved CX_7C motif suggest a role in thiol chemistry. A) Multiple amino acid sequence alignment of representative DUF1684 family members. UniProt reference numbers to left (D4GVL9 corresponds to HVO_1043). Orange highlight, residues > 95% sequence threshold. Bar above alignment colored red to blue, indicates regions of high to low sequence conservation, respectively. B) 3D-structural model of HVO_1043 represented as a ribbon (left) and surface (right) diagram. The C-terminal tail (C-tail) and conserved cysteine residues (C150 and C158) are indicated. HVO_1043 (96% of the sequence, 178 residues) was modeled with 100% confidence by the single highest scoring template: the NMR solution structure of *Haloarcula marismortui* rrnAC0354 (PDB: 2LNU). The NMR solution structure of *Halobacterium salinarium* VNG_0733h (PDB: 2LOK*)* was an additional high scoring (100% confidence) template.
