# Supplementary figures and images for: TrmB Family Transcription Factor as a Thiol-Based Regulator of Oxidative Stress Response
Source: mBio. 2022 Jul 20;13(4):e00633-22. doi: 10.1128/mbio.00633-22 (PMC9426492; doi:10.1128/mbio.00633-22)

A.

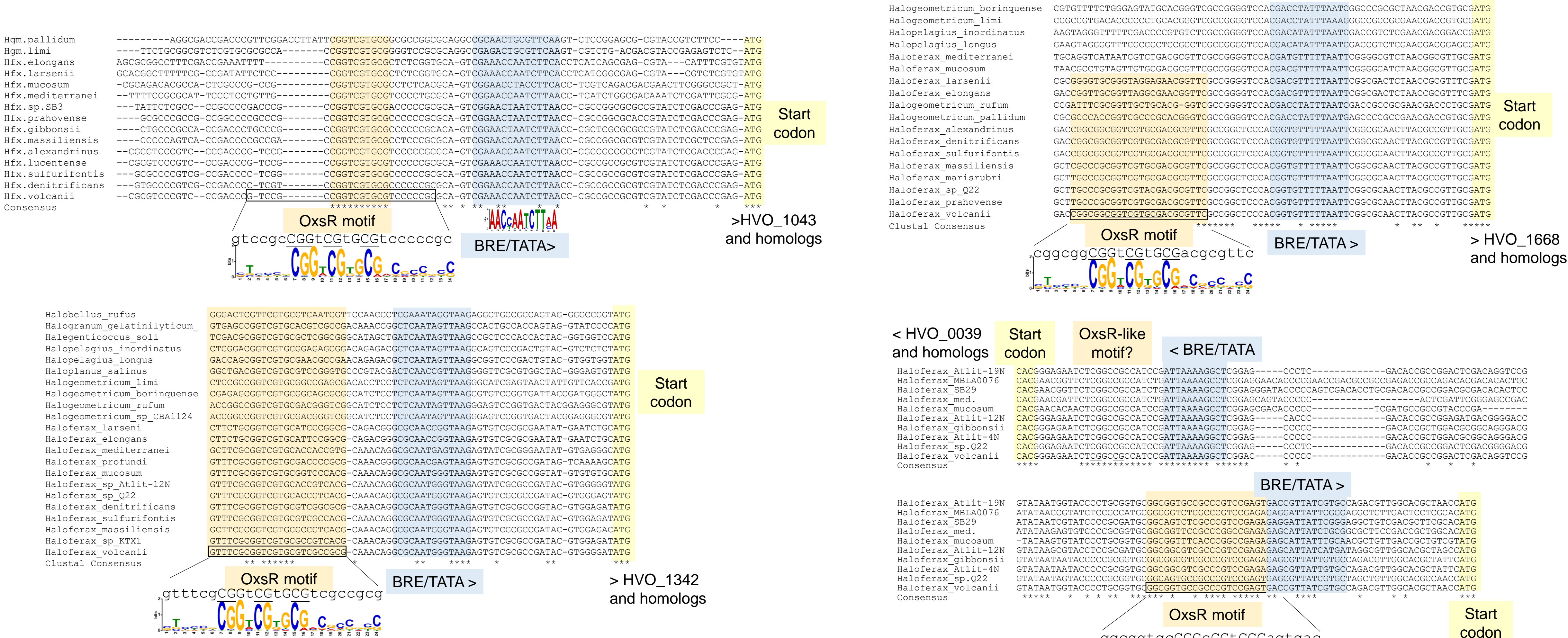

B.

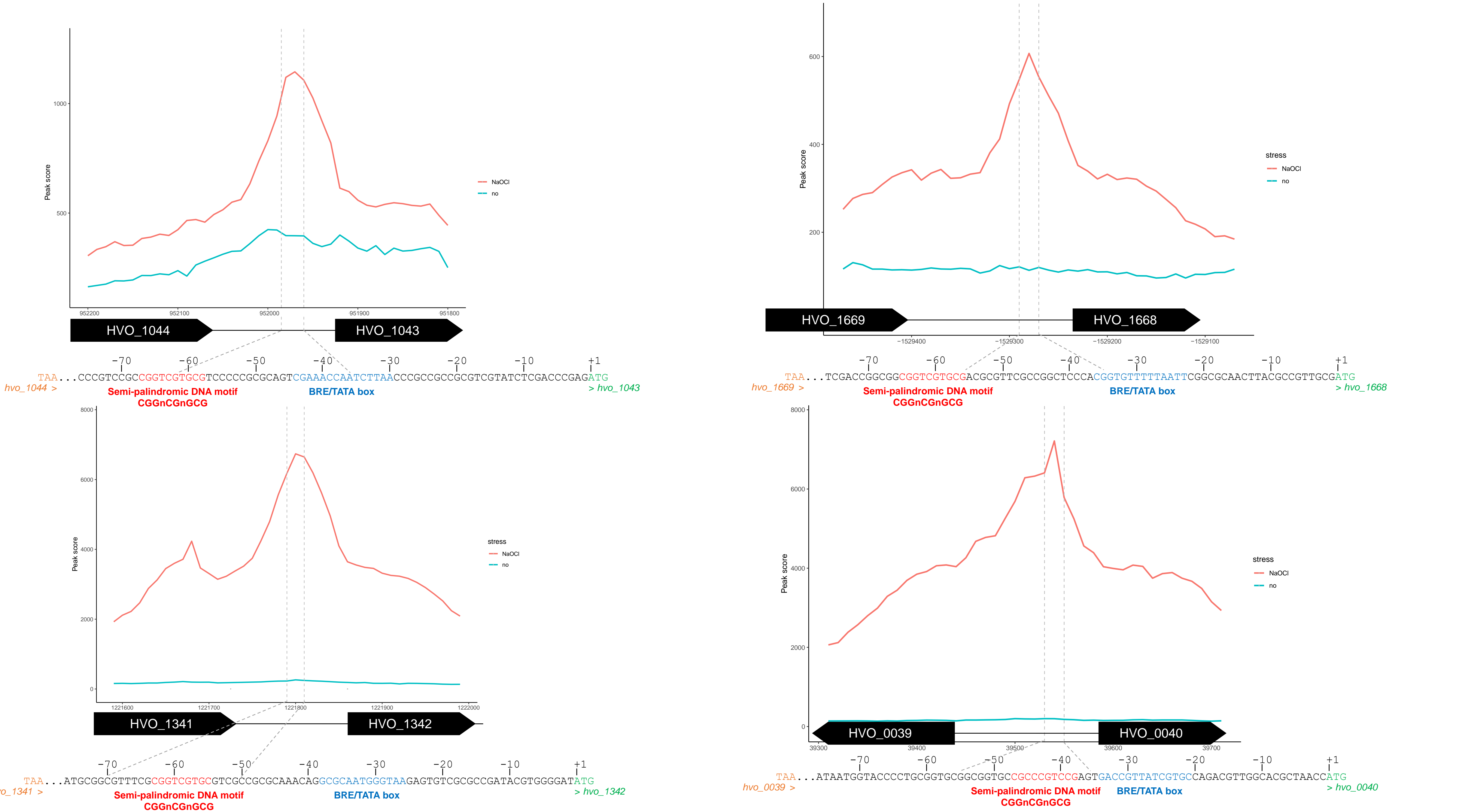

Supplement: FIG S5 [file mbio.00633-22-s0008.pdf]
