# Supplementary material for: TrmB Family Transcription Factor as a Thiol-Based Regulator of Oxidative Stress Response
Source: mBio. 2022 Jul 20;13(4):e00633-22. doi: 10.1128/mbio.00633-22 (PMC9426492; doi:10.1128/mbio.00633-22)
Supplement: FIG S6 [file mbio.00633-22-s0009.pdf]

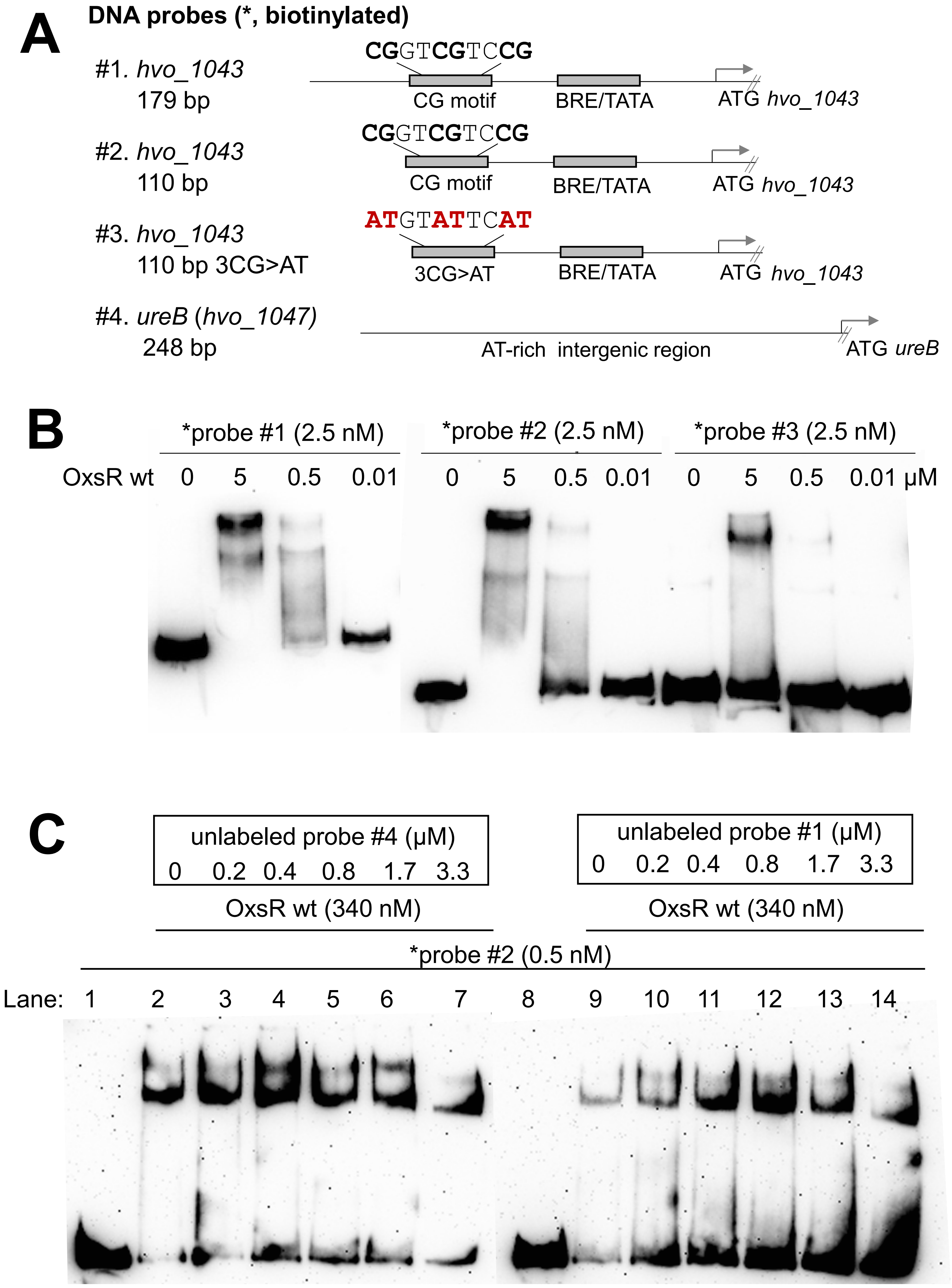

Figure S6. Analysis of OxsR binding to dsDNA by electrophoretic mobility shift assay (EMSA). A) Schematic of 5'-end biotin labeled (*) dsDNA probes used for EMSA. The dsDNA probe length (bp), CG-rich repeat (CG motif), conserved BRE/TATA promoter element, ATG start codon and gene (*hvo_1043* and *ureB*) 3' of the intergenic region carried on the probe as indicated. Probes #1 and 2 correlate with the 5' intergenic region of *hvo_1043* found bound to OxsR by ChIP-seq analysis. Probe #3 has site-directed modifications in the CG-rich repeat. Bold black is the wildtype DNA sequence and bold red is the CG>AT modified DNA sequence. (B) Comparison of OxsR binding to the dsDNA probes #1-3 by EMSA. OxsR was assayed at 0, 0.01, 0.5, 5 μM. C) Competition assay of OxsR binding to 5'-end biotin labeled probe #2. Probes #1 and 4 were unlabeled. Probe #1 is a specific competitor and probe #4 is a non-specific competitor. Our interpretation for the increased overall abundance of biotinylated probe detected as the specific competitor probe #1 increases (lanes 8 to 14) is as follows. We rule out loading error as the results are found experimentally reproducible and instead suggest not all conformations of OxsR bound to DNA enter the polyacrylamide gel and, thus, are not as readily detected at the low concentrations of specific competitor (probe #1) or at all concentrations of the non-specific competitor (probe #4). As the amount of specific competitor (probe #1) is increased in the reaction, the OxsR:DNA complexes are found to collapse and release free labeled probe that can now be separated and detected in the gel. For panels B and C, EMSA binding reactions (12 μl) were separated by 9 to 10% PAGE in 0.5×TBE buffer pH 8.3 after incubation with 1% formaldehyde for 10 min at room temperature in 50 mM HEPES, pH 7.5, 2 M NaCl, 10% glycerol, 15 mM $MgCl_2$, 1 mM EDTA supplemented with 0.25 μg/μL BSA and 0.1 μg/μL sheered salmon sperm DNA as described in hEMSA methods section. Biotinylated (*) probe (0.5 or 2.5 nM) and unlabeled probe (0 to 3.3 μM) were included in the assay as indicated. OxsR protein concentration was based on the purified homodimer.
