# Supplementary material for: TrmB Family Transcription Factor as a Thiol-Based Regulator of Oxidative Stress Response
Source: mBio. 2022 Jul 20;13(4):e00633-22. doi: 10.1128/mbio.00633-22 (PMC9426492; doi:10.1128/mbio.00633-22)
Supplement: FIG S7 [file mbio.00633-22-s0010.pdf]

A

28% amino acid identity

```
HVO_1360 --MASSMVEYL-QSDMECEGLLECLHGLKQLDRRCFEVLVETDDRLTVDEVAEAVERERS 57
OxsR     MADAPDMGELMETEDPNFGQVLACVFGIQSHESRTYLALLDN-PGSTVAELAEVLDRDRS 59
            *  .* *  :   .* :    :* *:.*::. : *  :  .*::.      **  *:**.::*:**

HVO_1360 TAYRSIQRLLQAGLIQKQQVNYEHGGYYHVYHPTDPNEVADDMQRMLNDWYAQMGTLIQE 117
OxsR     NVNRSLTTLLDKGLTERKRRLLDPGGYVYQYTATPLPEAKEMMHAALDEWAEDVHARIDA 119
         .. **:  **: ** ::::    : *** : *  *    *. : *:   *::*  :: : *:

HVO_1360 FRDKYDEKIVPAE 130
OxsR     FGES--------- 123
         * :.
```

B

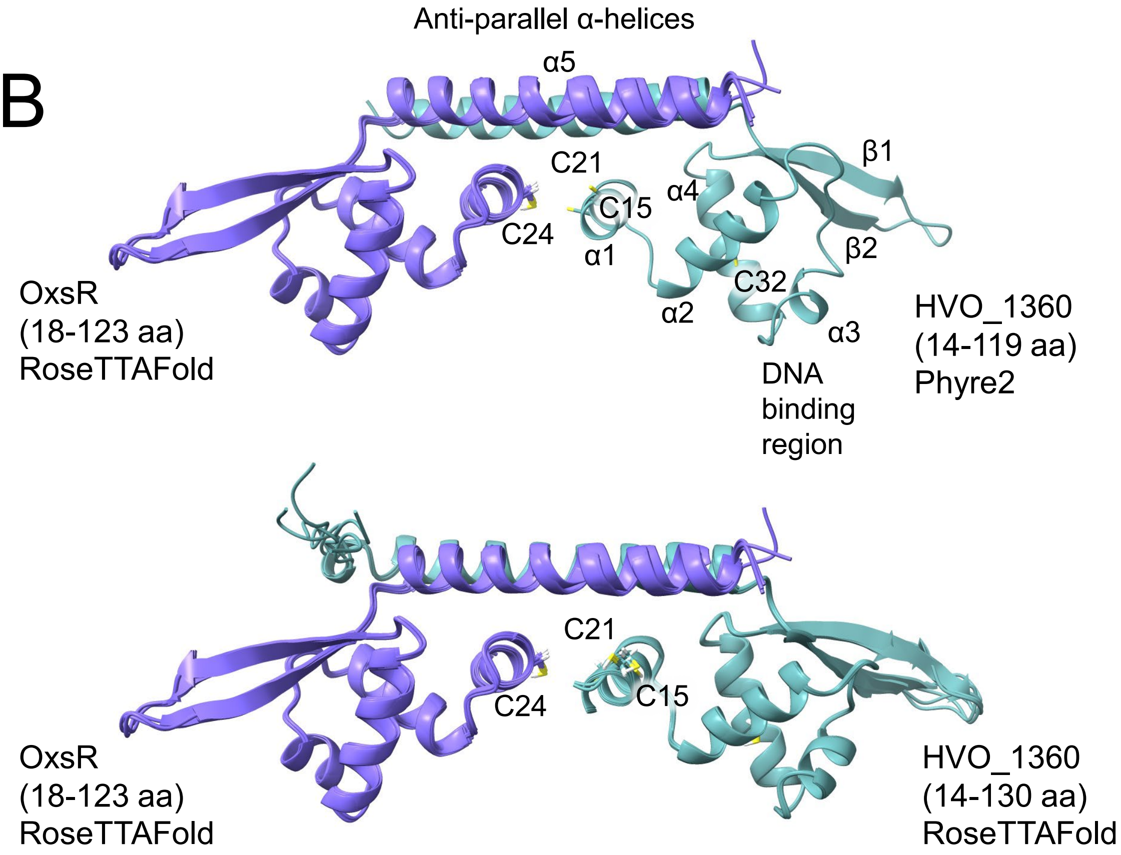

Figure S7. TrmB family proteins HVO_1360 and OxsR (HVO_2970) are structurally related. A) Amino acid sequence alignment of HVO_1360 and OxsR with identical (*), functionally similar (. or :), cysteine (red), and predicted DNA binding residues (purple) indicated. B) 3D-structural models of HVO_1360 (cadet green) and OxsR (purple). The monomeric 3D-structures were oriented as a heterodimer using *Sulfolobus acdiocaldarius* AbfR2 (PDB: 6CMV) as a scaffold. Models are presented as ribbon diagrams with cysteine residues as stick diagram and were performed by RoseTTAFold and Phyre2 as indicated. The N-terminal residues of OxsR (1-17 aa) and HVO_1360 (1-13 aa) which appeared variable in the 3D-models were manually removed. The OxsR and HVO_1360 Phyre2 models were related at an RMSD of 2.212 Å across all 106 atom pairs.
