# Supplementary material for: TrmB Family Transcription Factor as a Thiol-Based Regulator of Oxidative Stress Response
Source: mBio. 2022 Jul 20;13(4):e00633-22. doi: 10.1128/mbio.00633-22 (PMC9426492; doi:10.1128/mbio.00633-22)
Supplement: TABLE S1 [file mbio.00633-22-s0002.pdf]

Table S1. Representative TrmB family (PF01978) proteins of arCOG02242[1].

Protein	Organism	pI/Mr (kDa)	CC	Cysteine(s)	Phosphosites	Description(s)	Structure	Refs.
OxsR (HVO_2970)	*H. volcanii*	4.46/ 13.7	none	C24 required for activity; intersubunit (C24-C24) disulfide bond predicted	Y88, T91 conserved	mutant impaired in growth in the presence of hypochlorite; protein and transcript abundance upregulated by hypochlorite; transcriptional repressor/activator of genes associated with redox stress	3D-model	This study
HVO_1360	*H. volcanii*	4.57/ 15.3	α1, α4, α5	C15, C21, C32	Not conserved	Other *H. volcanii* member of this arCOG group	3D-model	This study
MM_1094	*Methanosarcina mazei*	7.71/ 14.0	none	intersubunit (C6-C17) disulfide bonds	Y83 conserved	Homodimer; subunit configuration appears altered in X-ray crystal structure	PDB: 3R0A	Protein Data Bank
Smj12 (SSO0458)	*Saccharolobus solfataricus*	9.35/ 12.9	α5	C105	Not conserved	Non-specific DNA binding; stabilizes double helix; introduces positive supercoiling; not abundant		(1)
Ss-Lrs14 (SSO1108)	*Saccharolobus solfataricus*	7.72/ 14.1	none	C25, C110	Y89 conserved	Homodimer; autorepressor; binds *adh* (alcohol dehydrogenase) promoter; generally displays large footprint; accumulates in late growth phases		(2-4)
AbfR2 (Saci_1223)	*Sulfolobus acdiocaldarius*	8.69/ 14.6	α1, α4, α5	None	Y88 conserved	Mutant impaired in biofilm formation; upregulated at transcript level during biofilm	PDB: 6CMV	(5-7)
Sa-Lrs14 (Saci_1242)	*Sulfolobus acdiocaldarius*	8.64/ 13.5	none	C15, C16, C26, C100	Y81, T84 conserved	Mutant impaired in biofilm formation; upregulated at transcript level during biofilm		(6, 7)
AbfR1 (Saci_0446)	*Sulfolobus acidocaldarius*	8.91/ 13.2	α5	C105	Demonstrated Y84(p), S87(p); phosphorylation important for DNA binding	Mutant: non-motile, increased extracellular polymeric substance (EPS) production, robust biofilm structure, upregulation of adhesive pili (*aap*), downregulation of archaellum (*fla*); AbfR1 binds *aap* and *fla* promoter regions in vitro; role as transcriptional		(6, 8)

						activator and repressor; upregulated at transcript level during biofilm		
Sto12a (ST1889)	*Sulfurisphaera tokodaii*	9.1/ 12.5	α5	intersubunit (C15-C15) intrasubunit (C16-C100) disulfide bonds	Y81, S84 conserved	Homodimer; α5 antiparallel coiled-coil homodimer interface	PDB: 2D1H	(9)

[1]CC, coiled-coil region predicted by DeepCoil (10). *Sulfolobus acidocaldarius* arCOG02242 members: Saci_0133, Saci_0102, Saci_1242 (Lrs14), Saci_1223 (AbfR2), Saci_0446 (AbfR1), Saci_1219.

Table S1 References

1. Napoli A, Kvaratskelia M, White MF, Rossi M, Ciaramella M. 2001. A novel member of the bacterial-archaeal regulator family is a nonspecific dna-binding protein and induces positive supercoiling. J Biol Chem 276:10745-52.

2. Napoli A, van der Oost J, Sensen CW, Charlebois RL, Rossi M, Ciaramella M. 1999. An Lrp-like protein of the hyperthermophilic archaeon *Sulfolobus solfataricus* which binds to its own promoter. J Bacteriol 181:1474-80.

3. Bell SD, Jackson SP. 2000. Mechanism of autoregulation by an archaeal transcriptional repressor. J Biol Chem 275:31624-9.

4. Fiorentino G, Cannio R, Rossi M, Bartolucci S. 2003. Transcriptional regulation of the gene encoding an alcohol dehydrogenase in the archaeon *Sulfolobus solfataricus* involves multiple factors and control elements. J Bacteriol 185:3926-34.

5. Vogt MS, Völpel SL, Albers SV, Essen LO, Banerjee A. 2018. Crystal structure of an Lrs14-like archaeal biofilm regulator from *Sulfolobus acidocaldarius*. Acta Crystallogr D Struct Biol 74:1105-1114.

6. Orell A, Peeters E, Vassen V, Jachlewski S, Schalles S, Siebers B, Albers SV. 2013. Lrs14 transcriptional regulators influence biofilm formation and cell motility of *Crenarchaea*. ISME J 7:1886-98.

7. Koerdt A, Orell A, Pham TK, Mukherjee J, Wlodkowski A, Karunakaran E, Biggs CA, Wright PC, Albers SV. 2011. Macromolecular fingerprinting of *Sulfolobus* species in biofilm: a transcriptomic and proteomic approach combined with spectroscopic analysis. J Proteome Res 10:4105-19.

8. Li L, Banerjee A, Bischof LF, Maklad HR, Hoffmann L, Henche AL, Veliz F, Bildl W, Schulte U, Orell A, Essen LO, Peeters E, Albers SV. 2017. Wing phosphorylation is a major functional determinant of the Lrs14-type biofilm and motility regulator AbfR1 in *Sulfolobus acidocaldarius*. Mol Microbiol 105:777-793.

9. Shinkai A, Sekine S, Urushibata A, Terada T, Shirouzu M, Yokoyama S. 2007. The putative DNA-binding protein Sto12a from the thermoacidophilic archaeon *Sulfolobus tokodaii* contains intrachain and interchain disulfide bonds. J Mol Biol 372:1293-304.

10. Ludwiczak J, Winski A, Szczepaniak K, Alva V, Dunin-Horkawicz S. 2019. DeepCoil-a fast and accurate prediction of coiled-coil domains in protein sequences. Bioinformatics 35:2790-2795.
