# Supplementary material for: TrmB Family Transcription Factor as a Thiol-Based Regulator of Oxidative Stress Response
Source: mBio. 2022 Jul 20;13(4):e00633-22. doi: 10.1128/mbio.00633-22 (PMC9426492; doi:10.1128/mbio.00633-22)
Supplement: TABLE S2 [file mbio.00633-22-s0003.pdf]

Table S2. List of strains, plasmids, and primers used in this study.

Strain, plasmid or primer	Description[a]	Source or Ref.
**Strains:**		
E. coli		
TOP10	F⁻ *mcrA Δ*(*mrr-hsdRMS-mcrBC*) Φ80*lacZΔ*M15 *ΔlacX74 recA1 araD139 Δ*(*ara leu*) 7697 *galU galK rpsL* (Str^r) *endA1 nupG λ-*	Invitrogen
GM2163	F⁻ *ara-14 leuB6 fhuA31 lacY1 tsx78 glnV44 galK2 galT22 mcrA dcm-6 hisG4 rfbD1 rpsL136 dam13*::Tn*9 xylA5 mtl-1 thi-1 mcrB1 hsdR2*	New England Biolabs
H. volcanii		
DS70	wild-type isolate DS2 cured of plasmid pHV2	(1)
H26	DS70 *ΔpyrE2*	(2)
SH125	H26 ΔoxsR	This study
SH126	H26 *oxsR*:*HA* integrant	This study
PM012	H26 *oxsR C24A:HA* integrant	This study
PM057	H26 *Δhvo_1043*	This study
PM058	H26 Δ24 bp 5'-GTCCGCCGGTCGTGCGTCCCCCGC-3' CG-rich motif 5' of the BRE/TATA consensus sequence of *hvo_1043*	This study
PM059	H26 Δ10 bp 5' CGGTCGTGCG-3' CG-rich motif 5' of the BRE/TATA consensus sequence of *hvo_1043*	This study
**Plasmids:**		
pTA131	Ap^r; pBluescript II containing P*fdx-pyrE2*	(2)
pJAM809	Ap^r Nv^r; pJAM202c-derived, carries P2$_{rrn}$-*hvo_1862-strepII* (*KpnI* site upstream of StrepII coding sequence)	(3)
pJAM202c	Ap^r; Nv^r; pJAM202-derived control plasmid	(4)
pJAM3380	Ap^r; pTA131-derived, carries *oxsR* and ~700 bp flanking sequence (pre-deletion plasmid)	This study
pJAM3381	Ap^r; pJAM3380 with *ΔoxsR* (deletion plasmid)	This study
pJAM3388	Ap^r Nv^r; pJAM809 with *oxsR* complementation plasmid	This study
pJAM3389	Ap^r; pJAM3380 with *oxsR*-HA (integrant plasmid)	This study
pJAM3901	Ap^r, pJAM3389 with *oxsR*-HA C24A (integrant plasmid)	This study
pJAM3919	Ap^r; pTA131 carries *hvo_1043* and ~500 bp flanking sequence (pre-deletion plasmid)	This study
pJAM3920	Ap^r; pJAM3919 with *Δhvo_1043* (deletion plasmid)	This study
pJAM3921	Ap^r; pJAM3919 with ΔDNA binding motif 5' of *hvo_1043* (deletion plasmid)	This study
pJAM3922	Ap^r; pJAM3919 with ΔDNA binding motif CG repeat (deletion plasmid)	This study
pJAM4019	Ap^r Nv^r; pJAM809 derived carries P2$_{rrn}$-*hvo_2970-strepII* (OxsR-StrepII)	This study
pJAM4020	Ap^r Nv^r; pJAM809 derived carries P2$_{rrn}$-*hvo_2970-strepII* (OxsR C24A StrepII)	This study

**Primers:**		
1. preKO_HVO_2970_HindIII_F	5’ ATTACAAGCTTCTTCGACAACGAACTCGTGA 3’	This study
2. preKO_HVO_2970_XbaI_R	5’ TAGTTTCTAGAGTAGCTGCCGTAGTCCTCGT 3’	This study
3. KO_HVO_2970_FW	5’ GCTCGCGGCCGACCG 3’; deletion HVO_2970	This study
4. KO_HVO_2970_RV	5’ GCACACCTGTTCGCCGTG 3’; deletion HVO_2970	This study
5. CompleHVO_2970_NdeI_F	5’ GTTACATATGGCCGACGCACCGGACATG 3’	This study
6. CompleHVO_2970_KpnI_R	5’ GATTAGGTACCCTACGACTCGCCGAAGGCGT 3’	This study
7. TrmBLHAtag_F	5’ CCCGGACTACGCCTAGGCTCGCGGCCGACCG 3’; integrate HVO_2970 HA tag	This study
8. TrmBLHAtag_R	5’ ACGTCGTACGGGTACGACTCGCCGAAGGCGTCG 3’; integrate HVO_2970 HA tag	This study
9. ext_HVO_2970_FW	5' GGCTCCGTACTACTTCGACA 3’	This study
10. ext_HVO_2970_RV	5' TCTCGATAGCTTCGACCATC 3’	This study
11. HAoxsR_C24A3 FW	5’ ACAGGTCCTCGCGGCGGTCTTCGGCATCC 3’	
12. HAoxsR_C24A3 RV	5’ GGATGCCGAAGACCGCCGCGAGGACCTGT 3’	This study
13. Qset2 HVO_2970_F	5’ AACTTCGGACAGGTCCTC 3’; qRT PCR HVO_2970 (*oxsR*)	This study
14. Qset2 HVO_2970_R	5’ TTGTCGAGTAGTGCGAGATA 3’; qRT PCR HVO_2970 (*oxsR*)	This study
15. rpl16 qPCR FW1 (forward primer)	5’-GCGAGTACATCACGGGTATC-3’	This study
16. rpl16 qPCR RV1 (reverse primer)	5’ CACTTCCTCTTCGACCTTCAG 3’	This study
17. HVO_0040 Primer pair 6_F	5’ GTCGTCATGGGAGCGATGAT 3’	This study
18. HVO_0040 Primer pair 6_R	5’ GCGACGTGGATTGTGAAGC 3’	This study
19. HVO_0039 Primer pair 3_F	5’ GAGTTGGCGGAGTTGAAGGA 3’	This study
20. HVO_0039 Primer pair 3_R	5’ TCGAAAATCTCATCGGGCGT 3’	This study
21. HVO_0811 Primer pair 2_F	5’ CCTCTCTTCGATGTGCACCC 3’	This study
22. HVO_0811 Primer pair 2_R	5’ GGATTGGTGGCAAAGAACCG 3’	This study
23. HVO_0337 Primer pair 2_F	5’ CGCGAAGGTGAAGGACAAAC 3’	This study
24. HVO_0337 Primer pair 2_R	5’ GTCTGGCCGCTTACCTCTTC 3’	This study

25. HVO_1043 Primer pair 1_F	5’ GAAGGACCGCTATCTCGCTG 3’	This study
26. HVO_1043 Primer pair 1_R	5’ GTCGTCGTATTCCCGGAGTT 3’	This study
27. preKO1043HindIII500up	5’ TACAAGCTTCGGCGAGTCCTGCTGGTTCGAG 3’	This study
28. preKO_HVO_1043_XbaI-507down	5’ ATTTCTAGACGTTCGCCCACCTCGATCACCTCGTCG 3’	This study
29. KO_HVO_1043_FW	5’ CCGAAGCCGAGAACTGAGAGACGCG 3’	This study
30. KO_HVO_1043_RV	5’ CTCGGGTCGAGATACGACGCGGCGG 3	This study
31. KO_OxsRMotif1043_FW	5’ GCAGTCGAAACCAATCTTAACCC 3’	This study
32. KO_OxsRMotif1043_RV	5’ GGGTCGGGACGGGACGCGAAAAG 3’	This study
33. KO_OxsRMotif1043_F2	5’ TCCCCCGCGCAGTCGAAACCAATC 3’	This study
34. KO_OxsRMotif1043_R2	5’ GCGGACGGGTCGGGACGGGACG 3’	This study
35. HVO_2970 KpnI no stop	5’-CAGGTACCCGACTCcCCGAAGGCGTCGAT-3’	This study
36. 1043-probe-R2*	5’-biotin-GCGTGCGGAGGGCGTCGAGC-3’	This study
37. 1043-probe-R2	5’-GCGTGCGGAGGGCGTCGAGC-3’	This study
38. 1043-probe-FBwt	5’-TAAGCCCCGCCTCCACAGCCGTTC-3’	This study
39. 1043-probe-FAwt	5’-GTCCGCCGGTCGTGCGTCCCC-3’	This study
40. 1043-probe-FA4	5’-GTCCGCatGTatTGatTCCCCCGCGCAG-3’	This study
41. *ureB* F XbaI	5’- AATTCTAGACTGTCGGCGTTGGC-3’	This study
42. ureB R NdeI	5’-GGCATATGGACACACACCGAACG-3’	This study

[a]Ap^r, ampicillin resistance; Nv^r, novobiocin resistance; Str^r, streptomycin resistance. P2$_{rrnA}$, rRNA promoter used for gene expression; *oxsR*, TrmB-like HVO_2970; HA, C-terminal hemagglutinin derived epitope tag., *ribL* (internal standard); HVO_2970, *trmBL* renamed *oxsR*. Underlined nucleotides represent restriction enzyme cutting site. SDM, site-directed mutagenesis.

Table S2 References

1. Wendoloski D, Ferrer C, Dyall-Smith ML. 2001. A new simvastatin (mevinolin)-resistance marker from *Haloarcula hispanica* and a new *Haloferax volcanii* strain cured of plasmid pHV2. Microbiology 147:959-64.

2. Allers T, Ngo HP, Mevarech M, Lloyd RG. 2004. Development of additional selectable markers for the halophilic archaeon *Haloferax volcanii* based on the *leuB* and *trpA* genes. Appl Environ Microbiol 70:943-53.

3. Humbard MA, Zhou G, Maupin-Furlow JA. 2009. The N-terminal penultimate residue of 20S proteasome α1 influences its N^{α} acetylation and protein levels as well as growth rate and stress responses of *Haloferax volcanii*. J Bacteriol 191:3794-803.

4. Reuter C, Uthandi S, Puentes J, Maupin-Furlow J. 2010. Hydrophobic carboxy-terminal residues dramatically reduce protein levels in the haloarchaeon *Haloferax volcanii*. Microbiology-SGM:248-255.
