# Supplementary material for: TrmB Family Transcription Factor as a Thiol-Based Regulator of Oxidative Stress Response
Source: mBio. 2022 Jul 20;13(4):e00633-22. doi: 10.1128/mbio.00633-22 (PMC9426492; doi:10.1128/mbio.00633-22)
Supplement: FIG S3 [file mbio.00633-22-s0006.pdf]

A.

Network Stats

number of nodes:	48	expected number of edges:	13
number of edges:	30	PPI enrichment p-value:	4.8e-05
average node degree:	1.25		
avg. local clustering coefficient:	0.275		

your network has significantly more interactions than expected (what does that mean?)

Functional enrichments in your network

explain columns

Molecular Function (Gene Ontology)				
GO-term	description	count in network	strength	false discovery rate
GO:0015036	Disulfide oxidoreductase activity	5 of 12	1.53	0.00080
GO:0015035	Protein disulfide oxidoreductase activity	4 of 11	1.47	0.0053

Protein Domains and Features (InterPro)				
domain	description	count in network	strength	false discovery rate
IPR017937	Thioredoxin, conserved site	3 of 4	1.78	0.0374
IPR036249	Thioredoxin-like superfamily	5 of 25	1.21	0.0321

B.

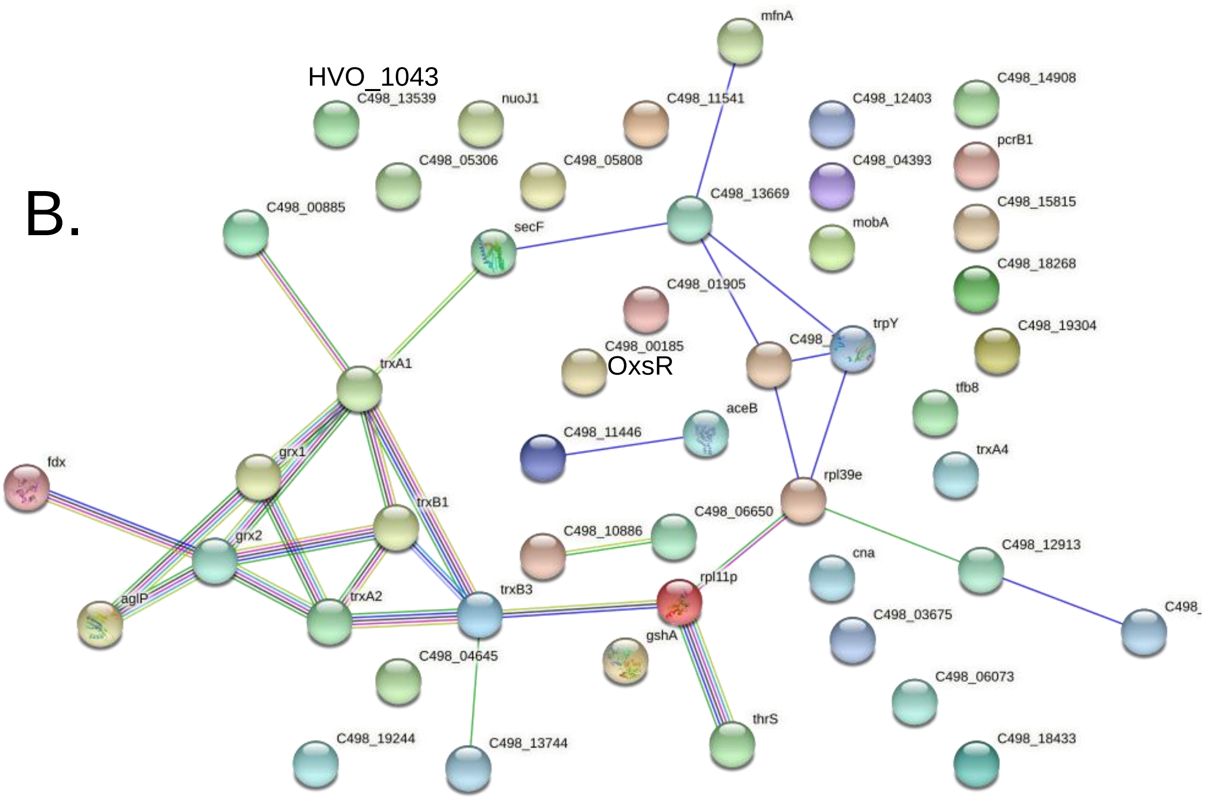

Figure S3. Functional enrichment of gene homologs associated with sulfur relay systems by STRING 11.5 analysis. OxsR and proteins encoded by genes immediately adjacent to OxsR-bound regions detected by ChIP-seq analysis analyzed by STRING (11.5) in tabulated (A) and node/edge configuration (B). Edges colored as: green line, neighborhood evidence; blue line, cooccurrence evidence; purple line, experimental evidence; yellow line, textmining evidence; light blue line, database evidence; black line, coexpression evidence. See **Dataset S1** for data input.
